# Isolation, Purification, Identification, and In Vitro ACE Inhibitory Activity of Tyrosine‐Arginine Dipeptide From Red Millet Huangjiu

**DOI:** 10.1002/fsn3.71749

**Published:** 2026-04-21

**Authors:** Jie Li, Junhui Zhong, Xin Zhang

**Affiliations:** ^1^ Zhang Zhongjing College of Chinese Medicine, Nanyang Institute of Technology Nanyang China

**Keywords:** ACE inhibitory peptide, fermented wine, identification, molecular docking, non‐competitive inhibition, red millet Huangjiu, separation and purification, tyrosine‐arginine

## Abstract

This study aims to isolate and identify endogenous peptides with angiotensin‐converting enzyme (ACE) inhibitory activity from red millet Huangjiu and to systematically elucidate their molecular mechanisms of action. Utilizing ultrafiltration, gel chromatography, and reversed‐phase high‐performance liquid chromatography (RP‐HPLC) followed by electrospray ionization tandem mass spectrometry (ESI‐MS/MS) analysis, the tyrosine‐arginine dipeptide (Tyr‐Arg) was successfully identified. In vitro assays demonstrated that Tyr‐Arg possesses moderate ACE inhibitory activity, with a half‐maximal inhibitory concentration (IC_50_) value of 158.93 ± 0.39 μM. Moreover, molecular docking analysis revealed that Tyr‐Arg binds to ACE via hydrogen bonding, π‐alkyl interactions, and van der Waals forces. Notably, the binding site was located outside the catalytic core, suggesting that Tyr‐Arg functions as a non‐competitive or allosteric inhibitor by inducing conformational changes in the enzyme. This mechanism was further corroborated by molecular dynamics (MD) simulations, which showed that the binding triggers a transition to a more open enzyme state, thereby obstructing substrate recognition or product release. MD simulations, integrated with molecular mechanics/generalized born surface area (MM/GBSA) calculations, substantiated that the Tyr‐Arg/ACE complex underwent significant structural expansion, as evidenced by the increased radius of gyration (*Rg*) and solvent‐accessible surface area (SASA), which stabilized after 80 ns in the late stage of the simulation. Despite the extensive expansion and the consequent high root mean square deviation (RMSD), the complex achieved a stable binding conformation during the final 20 ns, yielding a total binding free energy of −7.88 kcal/mol. The binding mechanism is primarily driven by a synergy of non‐polar interactions and electrostatic attraction; however, the electrostatic component is notably offset by the energetic costs associated with polar desolvation. Additionally, free energy landscape (FEL) analysis revealed the step‐wise sequential conformational transition pathway, identifying the global energy minima that stabilize the complex and prevent ligand dissociation, even in the face of substantial structural rearrangements observed during the 100 ns simulation. This study elucidates the molecular basis of ACE inhibition by Tyr‐Arg, providing a scientific foundation and novel insights for the valorization of functional components in traditional fermented foods.

## Graphical abstract

This study identified a novel ACE inhibitory dipeptide, Tyr‐Arg (YR), from red millet Huangjiu using multi‐step purification and ESI‐MS/MS. Tyr‐Arg exhibited effective inhibitory activity with an IC_50_ of 158.93 ± 0.39 μM. Molecular docking and 100‐ns molecular dynamics (MD) simulations revealed a non‐competitive inhibition mechanism, where Tyr‐Arg binds outside the catalytic core, inducing a conformational transition to a more “open” enzyme state. Structural expansion (increased *Rg* and SASA) and FEL analysis confirmed a stable binding energy (−7.88 kcal/mol) and a stepwise transition pathway, providing a molecular basis for the functional value of traditional fermented foods.

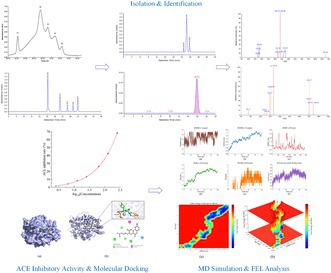

## 1 Introduction

Hypertension remains a prevalent chronic non‐communicable disease worldwide, posing a substantial threat to the health of middle‐aged and elderly populations (De Pascalis et al. 2024; Tran et al. 2022). A primary pathophysiological mechanism contributing to hypertension is the dysregulation of the renin‐angiotensin system (RAS), in which the angiotensin‐converting enzyme (ACE) serves as a pivotal regulatory component (Giani et al. 2021; Puspitojati et al. 2023). Although synthetic ACE inhibitors, such as captopril and enalapril, demonstrate potent clinical efficacy, their therapeutic application is frequently constrained by notable adverse effects, including angioedema and hyperkalemia, diminished efficacy with long‐term treatment (Ajayeoba et al. 2019; Jentsch Matias de Oliveira et al. 2020). Consequently, there is a pressing need to identify and develop safe, efficacious, and low‐side‐effect ACE inhibitors derived from natural sources.

Natural food‐derived bioactive peptides have garnered extensive attention owing to their high safety profile, ease of digestion and absorption, and diverse modes of action, such as competitive, non‐competitive, or mixed inhibition (Heo et al. 2020; Lee et al. 2019). Emerging evidence suggests that the fermentation processes of sake and grape wine produce bioactive peptides with significant ACE inhibitory activity. Peptidomic analyses have identified several critical ACE inhibitory sequences in sake and its byproduct, sake lees (*sake‐kasu*), including Val‐Val‐Tyr, Tyr‐Gly, and Phe‐Tyr. In addition to direct ACE inhibition, these peptides enhance vascular endothelial function by upregulating the endothelial nitric oxide synthase (eNOS)/nitric oxide (NO) signaling pathway (He et al. 2025). Similarly, mass spectrometry‐based investigations have identified bioactive sequences in grape wine, such as Leu‐Ser‐Pro‐His‐Pro and Val‐Asn‐Pro‐Ser‐Thr‐His‐Glu, which exert diverse regulatory effects (Fontana et al. 2025). These peptides derived from sake and grape wine exhibit a strong binding affinity for the ACE catalytic site.

Nanyang is a significant region for traditional Huangjiu production. Its unique geographical environment and climate have supported the cultivation of high‐quality winemaking raw materials, particularly red millet. Its red millet Huangjiu is produced from red millet sourced from southern Henan Province through multi‐strain co‐fermentation (Jin et al. 2021). Notably, red millet is rich in prolamins and albumins. During the fermentation of Huangjiu, proteases secreted by microorganisms facilitate the hydrolysis of these proteins into polypeptides and amino acids (He et al. 2013, 2024). These enzymatic degradation products may include functional peptide segments exhibiting ACE inhibitory activity. This study investigates Nanyang red millet Huangjiu to systematically elucidate the purification strategies, structural characteristics, and molecular mechanisms of its ACE inhibitory peptides. Based on our findings, we propose that the tyrosine‐arginine dipeptide (Tyr‐Arg) can interact with ACE at allosteric sites, modulating ACE activity via a non‐competitive inhibition mechanism.

## 2 Materials and Methods

### 2.1 Materials

Red millet Huangjiu was purchased from Runzhi Wine Co. Ltd. in Henan, China.

### 2.2 Chemicals

Anhydrous ethanol, hydrochloric acid, sodium hydroxide, sodium chloride, boric acid, sodium borate, and ammonium formate, all of analytical grade, were procured from China National Pharmaceutical Group Chemical Reagents Co. Ltd. Formic acid, trifluoroacetic acid, methanol, and acetonitrile, all of chromatographic grade, were obtained from Tianjin Kemiou Chemical Reagents Co. Ltd. ACE (EC 3.4.15.1, ≥ 2 units/mg protein), hippuric acid standard (≥ 98%), and N‐hippuryl‐L‐histidyl‐L‐leucine (HHL, ≥ 98%) were sourced from Sigma‐Aldrich Corporation, USA. Sephadex G‐15 dextran gel was acquired from Shanghai Hongshun Biotechnology Co. Ltd., China.

### 2.3 Preparation of Polypeptide Fractions From Red Millet Huangjiu

Briefly, 500 mL of the supernatant liquor was drawn from aged red millet Huangjiu and vacuum‐filtered utilizing a G4 sand core funnel in conjunction with a vacuum pump (SHB‐IIIS type circulating water multi‐purpose vacuum pump, Zhengzhou Great Wall Science and Technology Trade Co. Ltd., China). Next, powdered activated carbon (particle size approximately 0.1 mm) was mixed into the filtrate to reach a final concentration of 1% (w/v). A magnetic stirrer (Model 90‐1A, Shanghai Shuoguang Electronic Technology Co. Ltd., China) was then employed to stir the mixture at 300 rpm for 1 h at 25°C to facilitate decolorization. Following decolorization, the solution was filtered through a G4 sand core funnel to discard the activated carbon.

Thereafter, the decolorized filtrate was gradually introduced into anhydrous ethanol pre‐cooled to −20°C, and the final ethanol concentration was adjusted to 70% (v/v). The solution was thoroughly stirred and incubated at 4°C for 1.5 h to facilitate the precipitation of polysaccharides. Following incubation, it was centrifuged at 4°C and 8000 rpm for 20 min using a high‐speed refrigerated centrifuge (HERMLE Company, Germany), after which the supernatant liquor was obtained. Afterward, the supernatant liquor was concentrated using a rotary evaporator (RE‐52AA model, Zhengzhou Bio‐Chemical Instrument Co. Ltd., China) under vacuum conditions at 40°C and 0.07 MPa until its volume was reduced to approximately one‐third of the initial volume.

The concentrate was initially passed through a 0.22 μm membrane, followed by sequential ultrafiltration using a Pellicon‐2 ultrafiltration system (Millipore, USA). Ultrafiltration was initially performed using a 5 kDa membrane at 25°C under an operating pressure of 0.2 MPa to eliminate macromolecular substances. Subsequently, the 5 kDa filtrate was subjected to further separation using 3 kDa (0.15 MPa) and 1 kDa (0.1 MPa) ultrafiltration membranes in succession. The resulting filtrate, with a molecular weight below 1 kDa, and the retentate, with a molecular weight range of 1–3 kDa, were collected as peptide fractions with distinct molecular weight ranges. These two fractions were then lyophilized at −80°C utilizing a vacuum freeze‐dryer (Labconco, USA) to yield freeze‐dried crude peptides. The peptides were subsequently resuspended in ultrapure water to a concentration of 0.5 mg/mL for the assessment of their ACE inhibitory activity.

### 2.4 Determination of ACE Inhibitory Activity

#### 2.4.1 Construction of the Hippuric Acid Standard Curve

An accurately weighed 10.0 mg of hippuric acid standard was dissolved in ultrapure water. Subsequently, the solution was diluted to a final volume of 10 mL in a volumetric flask to yield a stock solution at 1.0 mg/mL (1000 μg/mL). From this stock solution, aliquots of 0, 25, 50, 100, 200, and 400 μL were pipetted into separate 2.0 mL centrifuge tubes. Each aliquot was subsequently diluted to a total volume of 1.0 mL using a 0.125 M HCl solution, resulting in hippuric acid standard solutions with concentration gradients of 0, 25, 50, 100, 200, and 400 μg/mL. High‐performance liquid chromatography (HPLC) was used to analyze each standard solution and record the corresponding peak areas. Lastly, a standard curve was generated by plotting hippuric acid concentration on the x‐axis and peak area on the y‐axis, following which linear regression analysis was performed (Tang et al. 2025).

#### 2.4.2 Assay of ACE Inhibition Rate

A solution of borate buffer with a pH of 8.3 was prepared, consisting of 0.125 M H_3_BO_3_ and 0.05 M NaCl. Then, HHL was accurately weighed and dissolved in the borate buffer solution to prepare a 10 mM stock solution (Feng et al. 2022). ACE was diluted using borate buffer to a working concentration of 0.15 U/mL. Afterward, a 1 M HCl solution was prepared for terminating the reaction (Ren et al. 2022).

Briefly, 50 μL of peptide solution (0.5 mg/mL), 50 μL of 10 mM HHL solution, and 200 μL of borate buffer were sequentially combined in a 2.0 mL centrifuge tube and thoroughly mixed. Next, the mixture was pre‐incubated at 37°C for 5 min, following which 50 μL of 0.15 U/mL ACE working solution was added, and the reaction was precisely carried out at 37°C for 30 min. Afterward, 50 μL of 1 M HCl was added to terminate the reaction, and the resulting mixture was thoroughly mixed. The total volume of the reaction system was 400 μL. Simultaneously, a blank control group (Control) was established, wherein an equal volume of borate buffer replaced the sample solution (Martin and Deussen 2019).

Finally, the terminated samples were centrifuged at 4°C and 12,000 rpm for 10 min, with 50 μL of the supernatant then collected for HPLC analysis.

#### 2.4.3 HPLC Analysis

The Agilent 1260 HPLC system (Agilent Technologies, USA), fitted with a ZORBAX Eclipse XDB‐C18 column (4.6 × 250 mm, 5 μm), was utilized for analysis. The mobile phase comprised Phase A, ultrapure water containing 0.05% (v/v) trifluoroacetic acid, and Phase B, acetonitrile containing 0.05% (v/v) trifluoroacetic acid. Isocratic elution was performed using a mixture of 75% Phase A + 25% Phase B. Chromatographic conditions were as follows: detection wavelength 228 nm, column temperature 25°C, flow rate 1.0 mL/min, and injection volume 10 μL.

The ACE inhibition rate (%) was calculated with the formula below:

$$\mathrm{ACE}\left(\%\right)=\frac{A_{\_\text{control}}-A_{\_\text{sample}}}{A_{\_\text{control}}}\times 100\%$$

where *A_control* refers to the concentration of hippuric acid in the blank control group (μg/mL) and *A_sample* represents the concentration of hippuric acid in the sample group (μg/mL).

### 2.5 Separation, Purification, and Identification of Red Millet Huangjiu Polypeptides

#### 2.5.1 Sephadex G‐15 Gel Filtration Chromatography

The < 1 kDa lyophilized peptide fractions were reconstituted in distilled water to a final concentration of 5 mg/mL. The solution was thoroughly vortexed to ensure complete dissolution and then filtered through a 0.22 μm membrane. The resulting filtrate was then loaded onto a Sephadex G‐15 gel column (dimensions: 16 mm × 70 cm; Dalian Yilite Analytical Instrument Co. Ltd., China) with a bed height of 50 cm. A loading volume of 3 mL was loaded. Distilled water was employed as the elution solvent, and isocratic elution was conducted at a flow rate of 0.5 mL/min. The eluent was collected using an automatic fraction collector (BSZ‐100, Shanghai Huxi Analytical Instrument Factory, China) at a rate of 1 mL per tube. UV absorbance was measured at a wavelength of 214 nm using an HD‐3 UV detector (Shanghai Huxi Analytical Instrument Factory, China). Based on the resulting elution profile, the fractions corresponding to each peak were accordingly combined. Then, the combined fractions were freeze‐dried utilizing a vacuum freeze‐dryer (Labconco Company, USA) to yield lyophilized fractions (Wang et al. 2021, 2020). Lastly, each lyophilized fraction was separately dissolved in ultrapure water to a final concentration of 0.5 mg/mL prior to the determination of its ACE inhibitory activity.

#### 2.5.2 Purification by Reversed‐Phase High‐Performance Liquid Chromatography (RP‐HPLC)

##### 2.5.2.1 Primary Purification

Following Sephadex G‐15 gel filtration chromatography, the most active freeze‐dried fraction was reconstituted in an aqueous solution of 20 mM ammonium formate with 0.1% (v/v) formic acid (pH 2.8) to a final concentration of 5 mg/mL, and then filtered through a 0.22 μm membrane. Primary purification was performed using an RP‐HPLC system (Agilent 1260) equipped with ZORBAX Eclipse XDB‐C18 column (4.6 mm × 250 mm, 5 μm). The injection volume was set to 5 μL. The mobile phase consisted of Phase A, an aqueous solution containing 20 mM ammonium formate and 0.1% (v/v) formic acid (pH 2.8), and Phase B, composed of acetonitrile containing 0.1% (v/v) formic acid. The gradient elution program was set as follows: 0–5 min, 5% B; 5–30 min, 5%–40% B; 30–40 min, 40%–95% B. Chromatographic conditions were maintained at a detection wavelength of 220 nm, a column temperature of 25°C, and a flow rate of 1.0 mL/min. The eluted peak fractions were collected, freeze‐dried, and separately redissolved in ultrapure water to a concentration of 0.5 mg/mL prior to ACE inhibitory activity assessment (Qiao et al. 2022; Wei et al. 2022).

##### 2.5.2.2 Secondary Purification

The most active freeze‐dried fraction from the primary purification step was reconstituted in mobile phase A (10 mM ammonium formate with 0.1% (v/v) formic acid in water, pH 2.8) to a final concentration of 1 mg/mL, and then filtered through a 0.22 μm membrane. The injection volume was 30 μL. The mobile phase consisted of an aqueous solution of 10 mM ammonium formate and 0.1% (v/v) formic acid (Phase A, pH 2.8) and acetonitrile with 0.1% (v/v) formic acid (Phase B). Gradient elution was performed as follows: 0–20 min, 5%–30% B; 20–40 min, 30%–65% B. Chromatographic conditions were maintained at a detection wavelength of 220 nm, a column temperature of 25°C, and a flow rate of 1.0 mL/min. The eluted peak fractions were collected, lyophilized, and separately reconstituted in ultrapure water to a concentration of 0.5 mg/mL prior to ACE inhibitory activity assessment (Wu et al. 2023; Zhou et al. 2023).

#### 2.5.3 Peptide Purity Analysis

The Agilent 1260 RP‐HPLC system equipped with a ZORBAX Eclipse XDB‐C18 column was used for purity analysis of the most active fraction obtained from secondary purification. The purified fraction was dissolved in mobile phase A (10 mM ammonium formate aqueous solution, pH 3.0) to prepare a 20 μg/mL solution. The injection volume was 5 μL. The mobile phase comprised Phase A (10 mM ammonium formate in water, pH 3.0) and Phase B (acetonitrile). The gradient elution program was as follows: 0–10 min, 5%–25% B; 10–30 min, 25%–60% B. Chromatographic conditions were maintained at a detection wavelength of 220 nm, a column temperature of 20°C, and a flow rate of 1.0 mL/min (Goyal et al. 2023; Zheng et al. 2020). Purity was calculated by peak area normalization.

#### 2.5.4 Identification of Purified Peptide via ESI‐MS/MS

The purified fraction was reconstituted in a 50% (v/v) acetonitrile (ACN) aqueous solution with 0.1% (v/v) formic acid (FA) to a final concentration of 5 μg/mL, and then filtered through a 0.22 μm membrane. Analysis was performed using electrospray ionization tandem mass spectrometry (ESI‐MS/MS) (Thermo Scientific TSQ Altis mass spectrometer, Thermo Fisher Scientific, USA). Mass spectrometry conditions were as follows: ionization mode: ESI^+^; mass scan range: *m*/*z* 50–700; resolution: unit resolution mode, approximately 0.7–1.0 Da FWHM; collision energy: 35 ± 5 eV; nebulizer gas (N₂) pressure: 40 psi; drying gas (N₂) flow rate: 10 L/min; ion source temperature: 300°C. Data acquisition was performed in data‐dependent acquisition (DDA) mode with a top N number of 10 and an MS/MS trigger threshold of 100 counts/s (Wei et al. 2025; Zeng et al. 2025).

### 2.6 Determination of Half‐Maximal Inhibitory Concentration (IC_50_ ) Value for ACE Inhibition by Purified Tyr‐Arg

Tyr‐Arg was dissolved in borate buffer to prepare stock solutions (20.00, 50.00, 100.00, 200.00, 500.00, 1000.00, and 2000.00 μM), yielding final reaction concentrations of 2.50, 6.25, 12.50, 25.00, 62.50, 125.00, and 250.00 μM, respectively. The ACE inhibitory activity was determined as described in Section 2.4. All assays were performed in triplicate, and the results were expressed as mean ± SD. A dose–response curve was constructed by plotting the mean ACE inhibition percentage (%) against the logarithm (log_10_) of the Tyr‐Arg concentration (μM). Nonlinear regression analysis was performed using Origin 2024 software (OriginLab Corp., Northampton, MA, USA) to fit the dose–response data, from which the IC_50_ was calculated (Jimsheena and Gowda 2009; Su et al. 2021).

### 2.7 Molecular Docking Analysis of Purified Tyr‐Arg With ACE

Molecular docking analyses were performed utilizing AutoDock 4.2, which employs a semi‐empirical free energy force field to evaluate binding affinities. The crystal structure of human ACE (PDB ID: 1O8A, resolution 2.00 Å) was retrieved from the Protein Data Bank. This structure was selected as it represents a high‐resolution C‐domain complexed with a dipeptide‐mimetic inhibitor (Enalaprilat), making it highly suitable for docking small dipeptides. Water molecules and co‐crystal ligands were removed using PyMOL (Schrödinger LLC), while *Zn* ^2+^ and Cl^−^ ions were retained in the active site. The receptor and ligand (Tyr‐Arg) were preprocessed by adding hydrogens and assigning Gasteiger charges using AutoDock Tools. The 3D structure of Tyr‐Arg was optimized using ChemOffice 2020 (PerkinElmer, USA). The docking grid box (76 Å × 90 Å × 94 Å) was centered on the *Zn* ^2+^ ion to encompass the entire active pocket. Conformational searching was conducted using the Lamarckian Genetic Algorithm (LGA). The binding energy was calculated as the sum of the intermolecular energy and the internal torsional energy. The conformation with the lowest binding free energy was selected for further visualization (Arámburo‐Gálvez et al. 2022; Dai et al. 2023; Duan et al. 2023).

### 2.8 Molecular Dynamics (MD) Simulations and Structural Stability Analysis of the Tyr‐Arg/ACE Complex

The optimal conformation with the lowest binding free energy from docking was selected as the initial model. A 100 ns MD simulation of the Tyr‐Arg/ACE complex was conducted using GROMACS (version 2023.2). The CHARMM36 all‐atom force field was employed for the entire system. The topology files for the protein and the dipeptide ligand were generated and subsequently merged to construct the complex system. The complex was solvated within a cubic periodic box using the TIP3P explicit water model, ensuring a minimum distance of 1.0 nm between the protein atoms and the box boundaries. Counterions were added to neutralize the net charge of the system. Energy minimization was performed using the steepest descent algorithm for a maximum of 50,000 steps. Subsequently, the system underwent a 5 ns equilibration in the canonical (NVT) ensemble, followed by a 5 ns equilibration in the isothermal‐isobaric (NPT) ensemble. During the NVT phase, the temperature was maintained at 298.15 K. In the NPT phase, the temperature and pressure were regulated at 298.15 K and 1.0 bar, respectively. Following equilibration, a 100 ns production MD trajectory was collected for subsequent analysis (Abraham et al. 2015; Huang and MacKerell Jr. 2013).

From the production trajectory, the root mean square deviation (RMSD), root mean square fluctuation (RMSF), radius of gyration (*Rg*), and solvent‐accessible surface area (SASA) were calculated and analyzed to evaluate the structural stability and conformational dynamics of the complex (Kumari et al. 2014). Binding free energies were quantified using the gmx_MMPBSA (v1.6.3) tool, which is based on MMPBSA.py (v16.0) (Homeyer and Gohlke 2012). All computational tasks were performed on the Beikun Cloud supercomputing platform. To comprehensively capture the energetic impact of the large‐scale conformational reorganization observed, a total of 100 snapshots were uniformly extracted from the entire 100 ns (0–100 ns) production trajectory for binding free energy calculations. This sampling strategy was chosen to account for the thermodynamic transition from the initial docking state to the final reorganized equilibrium. The receptor and ligand were defined using specific atom masks, corresponding to residues 1–574 (consistent with the RMSF profile) and the ligand (MOL), respectively. The solvation free energy was calculated using the Generalized Born (GB) model (igb = 2), and the non‐polar contribution to the solvation energy was determined via the Linear Combination of Pairwise Overlaps (LCPO) algorithm (Kumari et al. 2014; Lazaridis and Karplus 1999).

### 2.9 Statistical Analysis

All experiments were performed in triplicate, and data are presented as mean ± standard deviation (SD). Statistical analysis and graphical representation were conducted using Origin 2024 (OriginLab Corp., Northampton, MA, USA). Significant differences were determined via one‐way analysis of variance (ANOVA) followed by Tukey's post hoc test, with *p* < 0.01 considered statistically significant.

## 3 Results and Discussion

### 3.1 Standard Curve for Hippuric Acid

**FIGURE 1:**
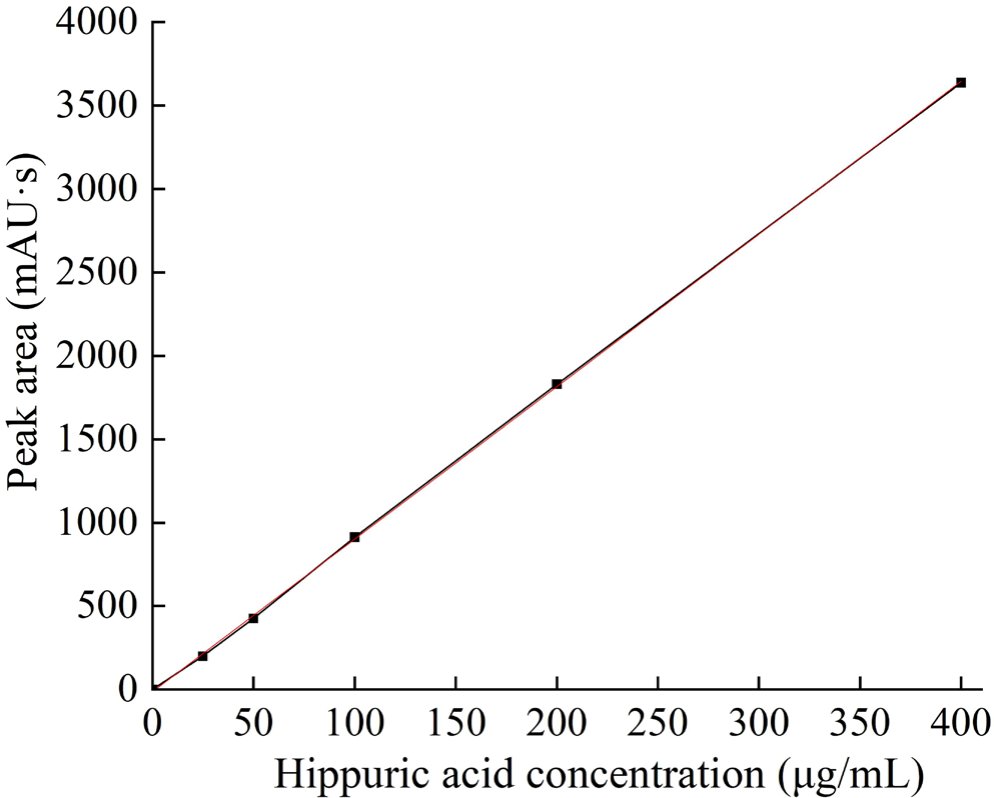
Standard curve of hippuric acid.

As illustrated in Figure 1, a strong linear relationship was noted between the concentration of hippuric acid and its HPLC peak area within the range of 0 to 400 μg/mL. The linear regression equation was *y* = 9.1438*x* − 13.4126 (*R* ^2^ = 0.999), indicating outstanding linearity within this concentration range. Thus, the standard curve was used for quantitative calculations of hippuric acid in subsequent ACE inhibitory rate determinations.

### 3.2 ACE Inhibitory Activity of Ultrafiltration Fractions With Different Molecular Weights

**FIGURE 2:**
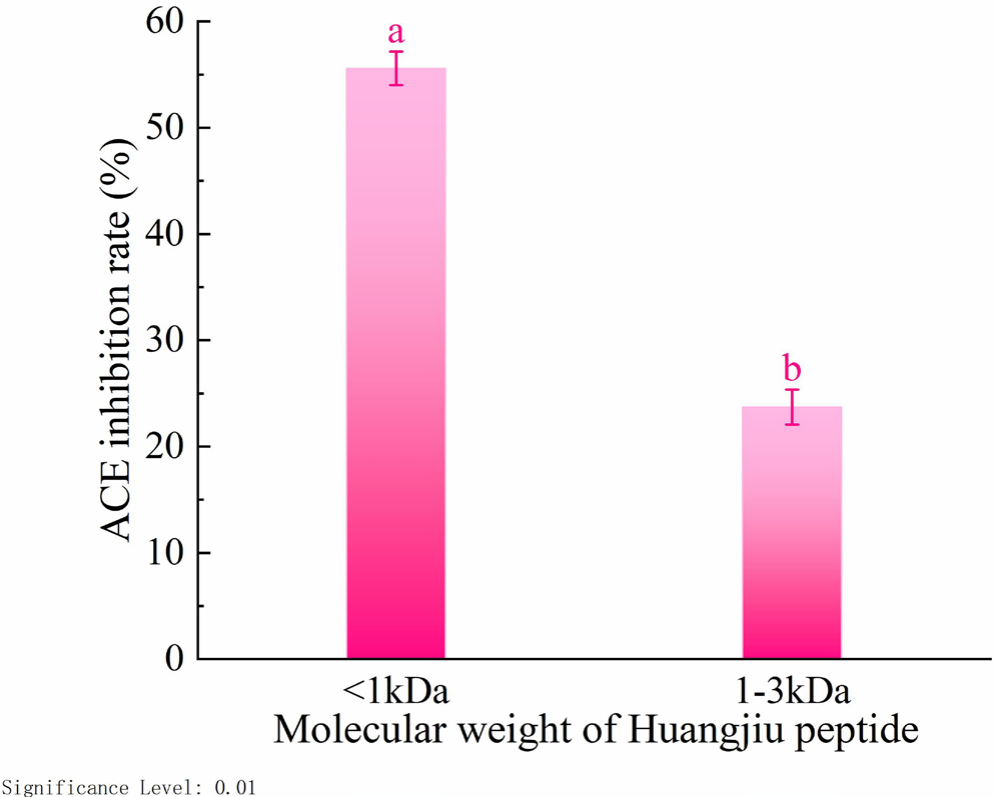
ACE inhibitory activity of ultrafiltration fractions with different molecular weights. Error bars represent the SD (*n* = 3). Different lowercase letters indicate significant differences (*p* < 0.01).

As displayed in Figure 2, the inhibitory activity of Huangjiu peptides against ACE varied across different molecular weight ranges. Among these fractions, the < 1 kDa fraction exhibited the highest ACE inhibitory rate of 55.59% ± 1.58%, which was significantly higher than that of the 1–3 kDa fraction (23.72% ± 1.65%, *p* < 0.01). This finding indicates that low‐molecular‐weight peptide segments may contain substantial amounts of fractions with significant ACE inhibitory activity (Choe et al. 2020; Patil et al. 2022). Consequently, the < 1 kDa fraction was selected for subsequent separation and purification.

### 3.3 Sephadex G‐15 Gel Filtration Chromatography Separation

**FIGURE 3:**
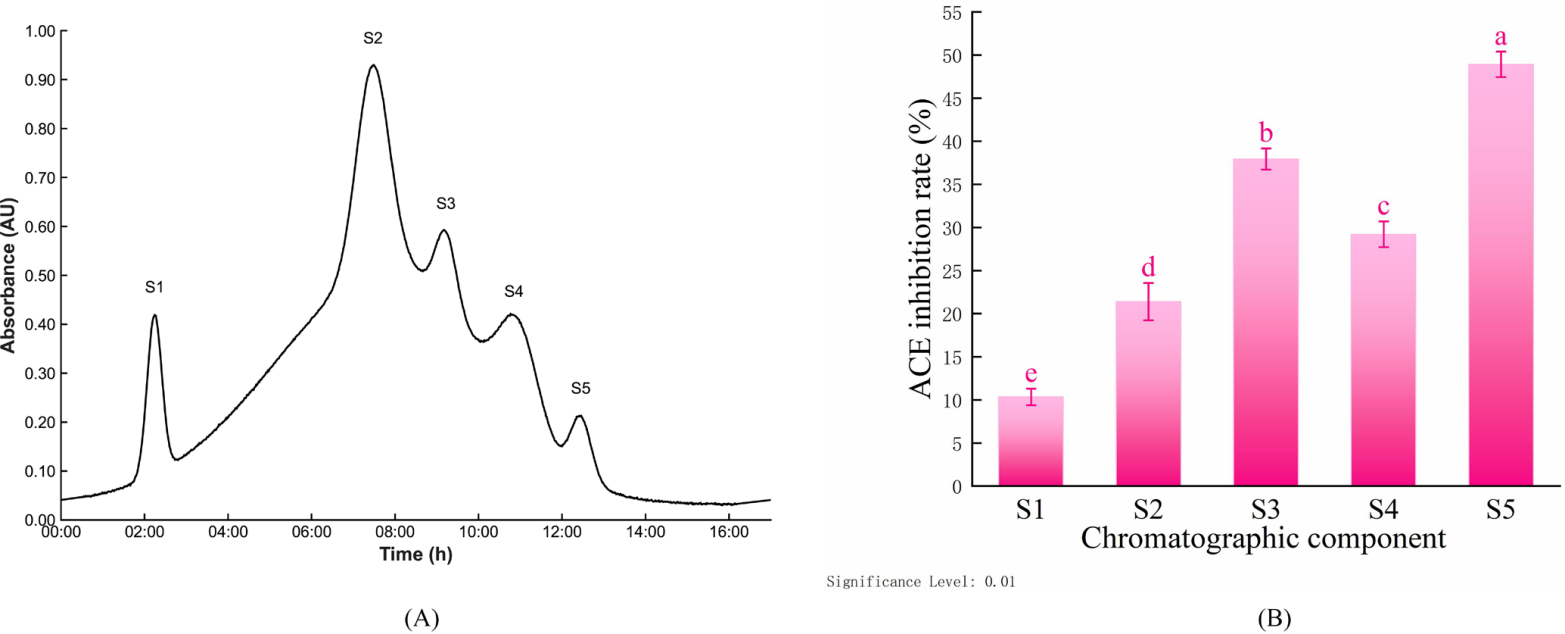
Sephadex G‐15 gel filtration chromatogram and ACE inhibitory activity of each fraction. (A) Sephadex G‐15 gel filtration chromatogram. (B) ACE inhibitory activity of each fraction. Error bars represent the SD (*n* = 3). Different lowercase letters indicate significant differences (*p* < 0.01).

As delineated in Figure 3A, the < 1 kDa fraction was separated using Sephadex G‐15 gel filtration chromatography, yielding five main elution peaks, labeled S1–S5. The elution times for each fraction were as follows: S1 (approximately 2.24 h), S2 (approximately 7.47 h), S3 (approximately 9.22 h), S4 (approximately 10.95 h), and S5 (approximately 12.45 h). In accordance with the principle of gel filtration chromatography, elution time is directly proportional to molecular weight (Li et al. 2022; Moayedi et al. 2018). Consequently, the S5 fraction comprised peptide segments with the lowest molecular weight.

As depicted in Figure 3B, all fractions exhibited significantly varying degrees of ACE inhibitory activity (*p* < 0.01). Among the examined fractions, the S5 fraction exhibited the highest inhibitory rate (48.92% ± 1.47%), which was significantly higher than the rates observed for the other fractions. Specifically, the inhibitory rates of fractions S3, S4, S2, and S1 were 37.94% ± 1.22%, 29.21% ± 1.50%, 21.40% ± 2.16%, and 10.34% ± 0.96%, respectively. These findings collectively suggest that the S5 fraction, which possesses the lowest molecular weight, is abundant in fractions with higher ACE inhibitory activity. The S5 fraction was thus selected for subsequent RP‐HPLC purification.

### 3.4 RP‐HPLC Purification

#### 3.4.1 Primary Purification

**FIGURE 4:**
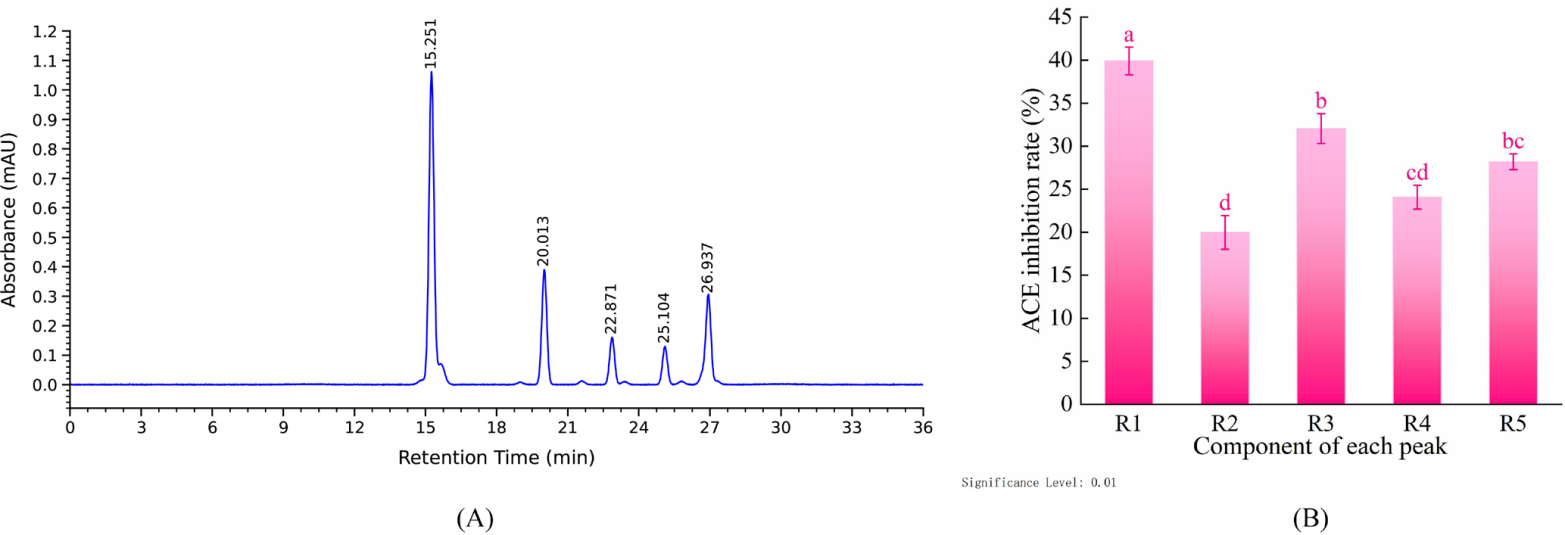
RP‐HPLC primary purification spectrum of S5 fraction and ACE inhibitory activity of each peak fraction. (A) RP‐HPLC primary purification spectrum of S5 fraction; (B) ACE inhibitory activity of each peak fraction. Error bars represent the SD (*n* = 3). Different lowercase letters indicate significant differences (*p* < 0.01).

As presented in Figure 4A, the S5 fraction was subjected to primary purification by RP‐HPLC, yielding five major peaks upon elution, designated R1–R5 according to their elution sequence. This result suggests that the S5 fraction is a multi‐component mixture. In accordance with the separation principle of RP‐HPLC, a direct correlation exists between the retention time of a fraction and its degree of hydrophobicity (Wei et al. 2019). The retention time of a substance is directly proportional to its degree of hydrophobicity. In other words, fractions with earlier retention times display weaker hydrophobicity, whereas those with later retention times exhibit stronger hydrophobicity (Gu et al. 2022). Consequently, it can be inferred that the R1 fraction exhibited comparatively lower hydrophobicity, whereas the R5 fraction manifested relatively higher hydrophobicity.

As demonstrated in Figure 4B, all fractions exhibited ACE inhibitory activity. Significant differences in activity were noted among the fractions (*p* < 0.01). Specifically, the R1 fraction exhibited the highest inhibitory rate (39.92% ± 1.61%), which was significantly higher than that of the other fractions (*p* < 0.01). The R3 (32.06% ± 1.74%) and R5 (28.20% ± 0.91%) fractions demonstrated relatively high activity; however, the difference between R4 (24.08% ± 1.39%) and R5 was not statistically significant. The R2 fraction exhibited the lowest level of activity (19.99% ± 1.95%), which was significantly lower compared to other fractions (*p* < 0.01). Collectively, these results suggest that the R1 fraction is enriched with high‐activity fractions. Thus, the R1 fraction was selected for secondary purification.

#### 3.4.2 Secondary Purification

**FIGURE 5:**
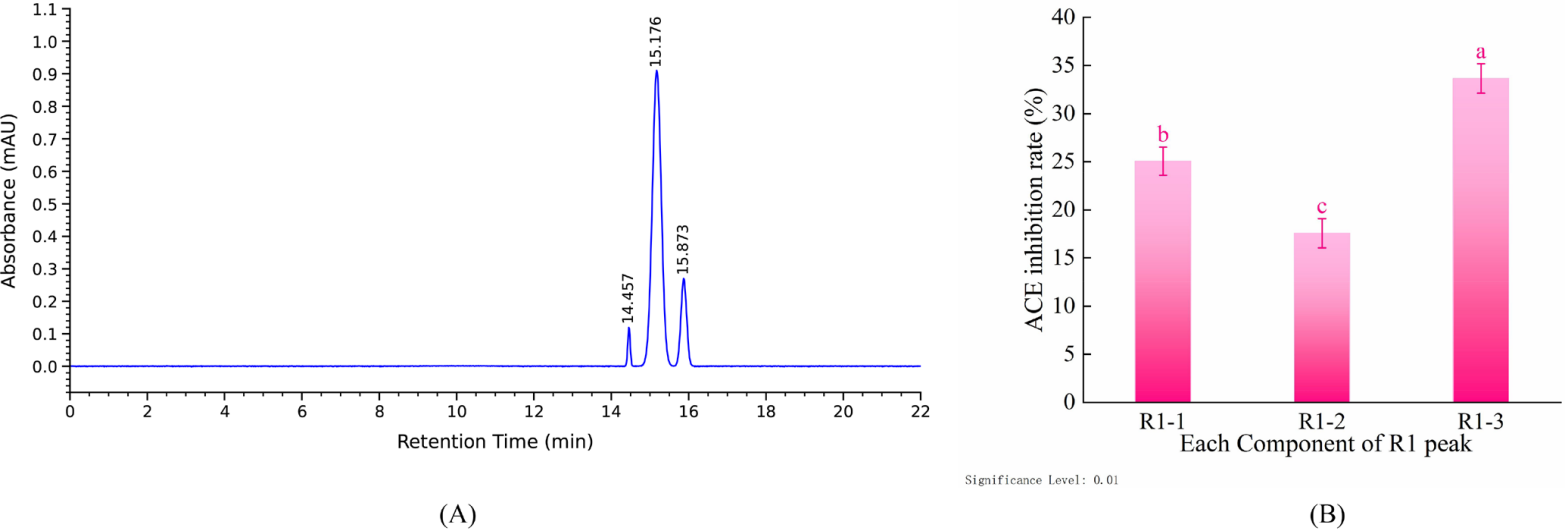
RP‐HPLC secondary purification spectrum of the R1 fraction and ACE inhibitory activity of each peak fraction. (A) RP‐HPLC secondary purification spectrum of the R1 fraction; (B) ACE inhibitory activity of each peak fraction. Error bars represent the SD (*n* = 3). Different lowercase letters indicate significant differences (*p* < 0.01).

As shown in Figure 5A, following secondary purification by RP‐HPLC, the R1 fraction underwent further separation into three primary peaks, designated R1‐1, R1‐2, and R1‐3. As displayed in Figure 5B, the ACE inhibitory rate of the R1‐3 fraction was the highest (33.66% ± 1.52%) and was significantly higher than that of the R1‐1 (25.06% ± 1.46%) and R1‐2 (17.59% ± 1.51%) fractions (*p* < 0.01).

### 3.5 ACE Inhibitory Activity of Peptide Fractions From Huangjiu at Different Purification Stages

The ACE inhibitory activities of Huangjiu peptide fractions at different purification stages are detailed in Table 1. The data indicate that the purification process effectively isolated and concentrated the bioactive constituents. Notably, the inhibition rate of the final fraction, R1‐3 (33.66% ± 1.52%), was lower than that of the initial < 1 kDa fraction (55.59% ± 1.58%). This reduction can be attributed to the synergistic effects among the multiple peptides present in the crude extract. As purification advanced, the disruption of these synergistic interactions resulted in a decline in overall inhibitory activity, despite the increased purity of individual peptides. Nonetheless, R1‐3 emerged as the most significant fraction obtained in the final purification stage and was consequently selected for further analysis of purity and structural identification.

**TABLE 1:** ACE inhibitory activity of different peptide fractions obtained during the purification process.

Purification stage	Fraction	ACE inhibition rate (%) (mean ± SD)
Ultrafiltration	< 1 kDa	55.59 ± 1.58
	1–3 kDa	23.72 ± 1.65
Sephadex G‐15	S1	10.34 ± 0.96
	S2	21.40 ± 2.16
	S3	37.94 ± 1.22
	S4	29.21 ± 1.50
	S5	48.92 ± 1.47
RP‐HPLC primary	R1	39.92 ± 1.61
	R2	19.99 ± 1.95
	R3	32.06 ± 1.74
	R4	24.08 ± 1.39
	R5	28.20 ± 0.91
RP‐HPLC secondary	R1‐1	25.06 ± 1.46
	R1‐2	17.59 ± 1.51
	R1‐3	33.66 ± 1.52

### 3.6 RP‐HPLC Purity Analysis of the Purified Fraction R1‐3

**FIGURE 6:**
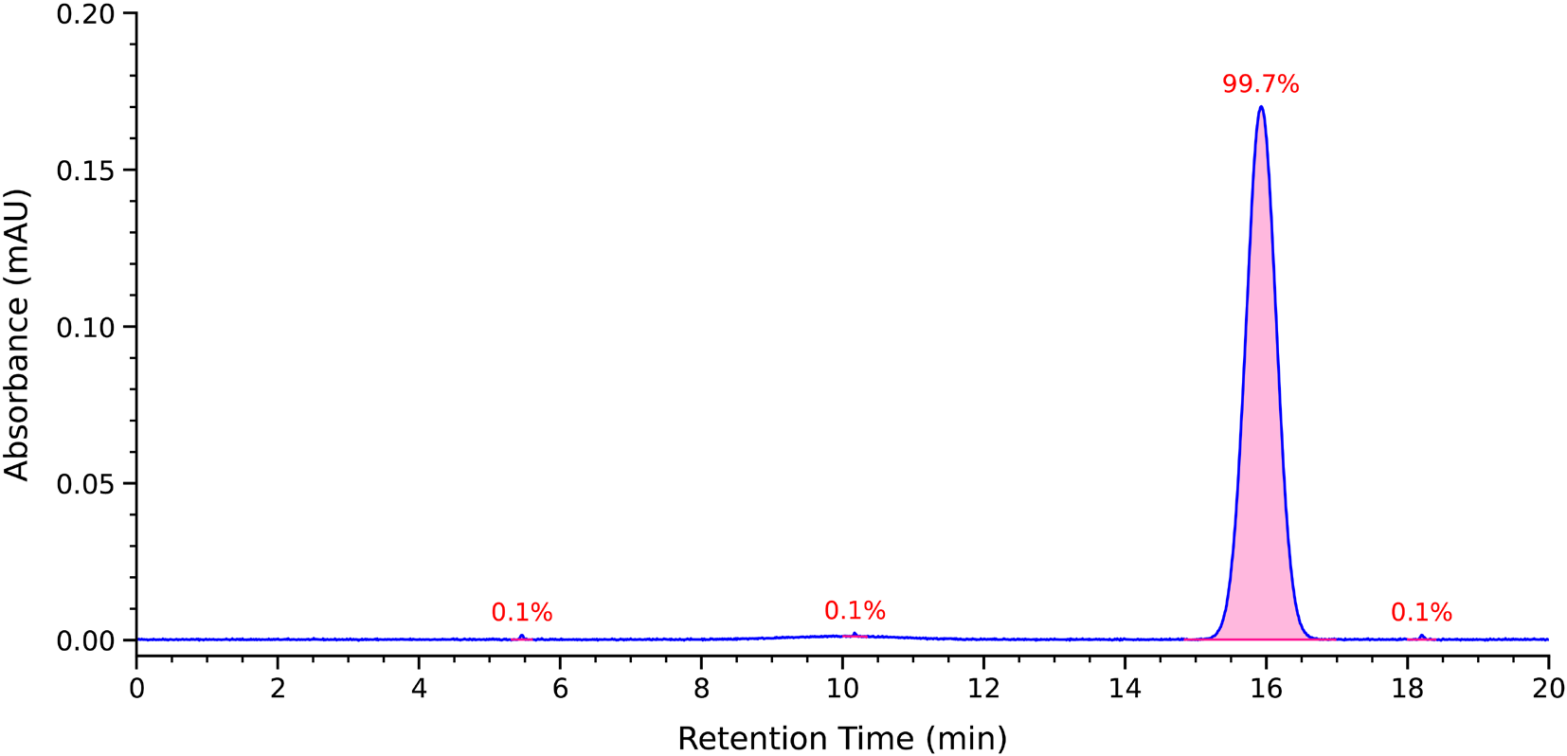
RP‐HPLC purity analysis of the purified fraction R1‐3.

As demonstrated in Figure 6, the RP‐HPLC chromatogram of the purified fraction R1‐3 unveiled a solitary symmetrical peak, suggesting a high degree of purity. The purity of the substance was determined to be 99.7% based on peak area normalization. This level of purity fulfills the criteria for subsequent mass spectrometric identification. Consequently, the structural identification of the purified R1‐3 fraction was conducted.

### 3.7 ESI‐MS/MS Identification of the Purified Fraction R1‐3

**FIGURE 7:**
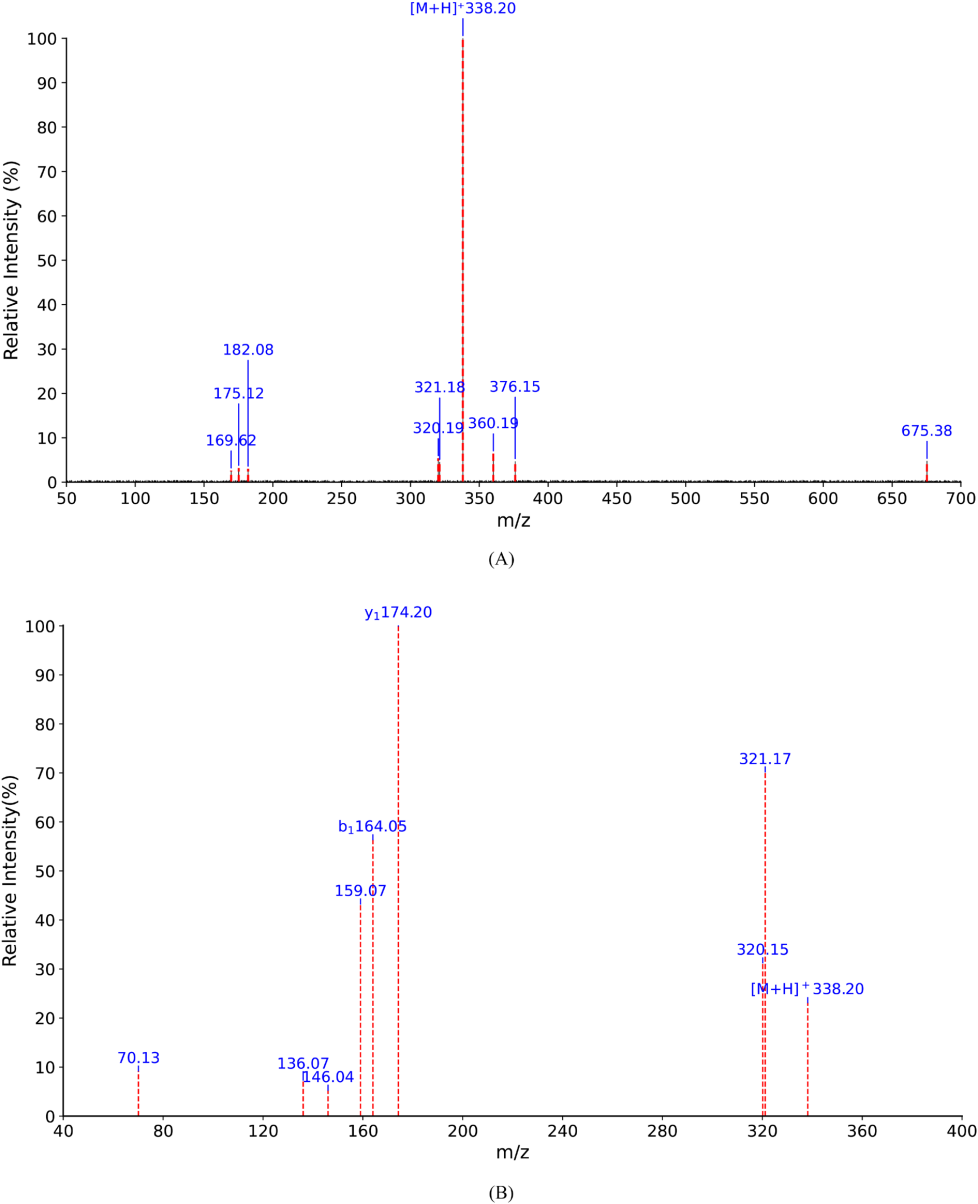
ESI‐MS/MS spectrum of the purified fraction R1‐3: (A) MS1 spectrum of the fraction R1‐3. (B) MS/MS spectrum of the fraction R1‐3 at *m/z* 338.20.

As demonstrated in Figure 7A, the primary mass spectrum of the purified fraction R1‐3 displayed the most intense mass‐to‐charge ratio signal at *m*/*z* 338.20, corresponding to the primary charged molecular ion peak of this fraction. Theoretically, the linear Tyr‐Arg has a molecular weight of 337.18 Da, and its protonated molecular ion [M + H]^+^ has a theoretical mass of 338.18 Da. As anticipated, the observed ion peak at *m*/*z* 338.20 closely matched the theoretical [M + H]^+^ mass of Tyr‐Arg (error 0.02 *m*/*z*), providing substantial evidence that this fraction is Tyr‐Arg.

Furthermore, tandem mass spectrometry (MS/MS) fragmentation analysis was performed on the precursor ion at *m*/*z* 338.20, with the results illustrated in Figure 7B. In MS/MS, peptide segments predominantly undergo cleavage along peptide bonds, yielding N‐terminal fragments (*b* ions) and C‐terminal fragments (*y* ions) (Chingin et al. 2014; Zhuang et al. 2016). For Tyr‐Arg dipeptide, the theoretical production of *b*_*1*_ and *y*_1_ ions is expected. As demonstrated in the MS/MS spectrum in Figure 7B, an ion peak at *m*/*z* 174.20 was identified in the fragmentation spectrum at *m*/*z* 338.20, which closely matched the theoretical *y*_1_ ion (an Arg residue, theoretical *m*/*z* 174.11) with an error of 0.09 *m*/*z*. This finding supports the C‐terminal amino acid as Arg. Additionally, an ion peak at *m*/*z* 164.05 was detected, which was consistent with the theoretical *b*_1_ ion (a tyrosine residue, theoretical *m*/*z* 164.07) with an error of 0.02 *m*/*z*. This finding provides further evidence that supports the N‐terminal amino acid as Tyr.

Notably, a high degree of consistency was observed between the mass of the precursor ion *m*/*z* 338.20 in the MS1 spectrum and the theoretical mass of Tyr‐Arg [M + H]^+^. Likewise, this consistency was observed between the characteristic fragment ions *m*/*z* 174.20 (*y*_1_) and *m*/*z* 164.05 (*b*_1_) noted in the MS/MS spectrum. Based on these observations, the purified peptide segment was precisely identified as Tyr‐Arg. Tyr‐Arg is a recognized ACE‐inhibiting peptide that has been identified in various protein hydrolysates and fermented foods. However, to the best of our knowledge, this is the first study to identify its presence in red millet Huangjiu.

### 3.8 Determination of IC_50_ of ACE Inhibitory Activity of Purified Tyr‐Arg

**FIGURE 8:**
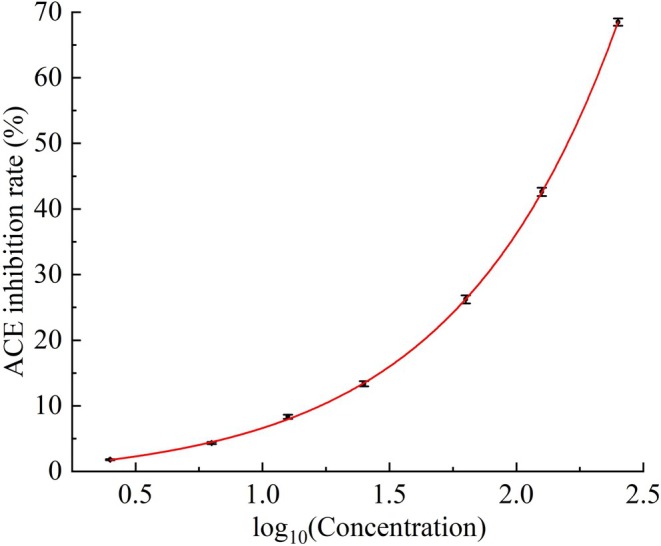
Dose–response relationship between the log_10_‐transformed concentration of Tyr‐Arg and its ACE inhibitory activity.

Following the procedure in Section 2.6, the ACE inhibitory activity of Tyr‐Arg was evaluated at various concentrations (Table 2). Tyr‐Arg exhibited a pronounced dose‐dependent inhibitory effect, with the inhibition rate increasing from (1.78% ± 0.08%) at 2.50 μM to (68.47% ± 0.56%) at 250.00 μM. The dose–response curve, illustrated in Figure 8, was generated by plotting the ACE inhibition rate (%) against the log_10_ of the Tyr‐Arg concentration.

**TABLE 2:** ACE inhibitory activity of Tyr‐Arg at different concentrations (*n* = 3).

Concentration (μM)	log_10_ (concentration)	Mean inhibition rate (%) (mean ± SD)
2.50	0.40	1.78 ± 0.08
6.25	0.80	4.34 ± 0.15
12.50	1.10	8.33 ± 0.30
25.00	1.40	13.37 ± 0.41
62.50	1.80	26.23 ± 0.63
125.00	2.10	42.59 ± 0.64
250.00	2.40	68.47 ± 0.56

As demonstrated in Figure 8, a nonlinear relationship was identified between the log_10_‐transformed Tyr‐Arg concentration and the ACE inhibition rate. Nonlinear regression analysis established a mathematical model describing this relationship as *y* = 1.7109e^1.5459*x*^ − 1.4145. The model's coefficient of determination (*R* ^*2*^) was 0.999, indicating an excellent goodness of fit to the experimental data.

Based on the established regression model, the IC_50_ value of Tyr‐Arg against ACE was determined to be 158.93 ± 0.39 μM. In comparison, the synthetic inhibitor captopril, employed as a positive control, exhibits significantly higher potency with IC_50_ values typically ranging from 0.005 to 0.05 μM. This marked disparity in inhibitory activity is consistent with previously reported data comparing natural peptides to synthetic drugs (Bhuyan and Mugesh 2011; Kim et al. 2016; Li et al. 2025). The superior potency of captopril is primarily attributed to its sulfhydryl (‐SH) moiety, which forms a high‐affinity coordination bond with the zinc ion (*Z*_*n*_^2+^) at the ACE catalytic site. Nevertheless, as a food‐derived dipeptide, Tyr‐Arg offers distinct advantages, including a superior safety profile and a lower risk of adverse effects. Consequently, it represents a promising candidate for long‐term blood pressure management through dietary intervention or the development of functional foods, providing a milder and potentially safer alternative to conventional pharmacological therapies.

### 3.9 Molecular Docking Study of Tyr‐Arg With ACE

**FIGURE 9:**
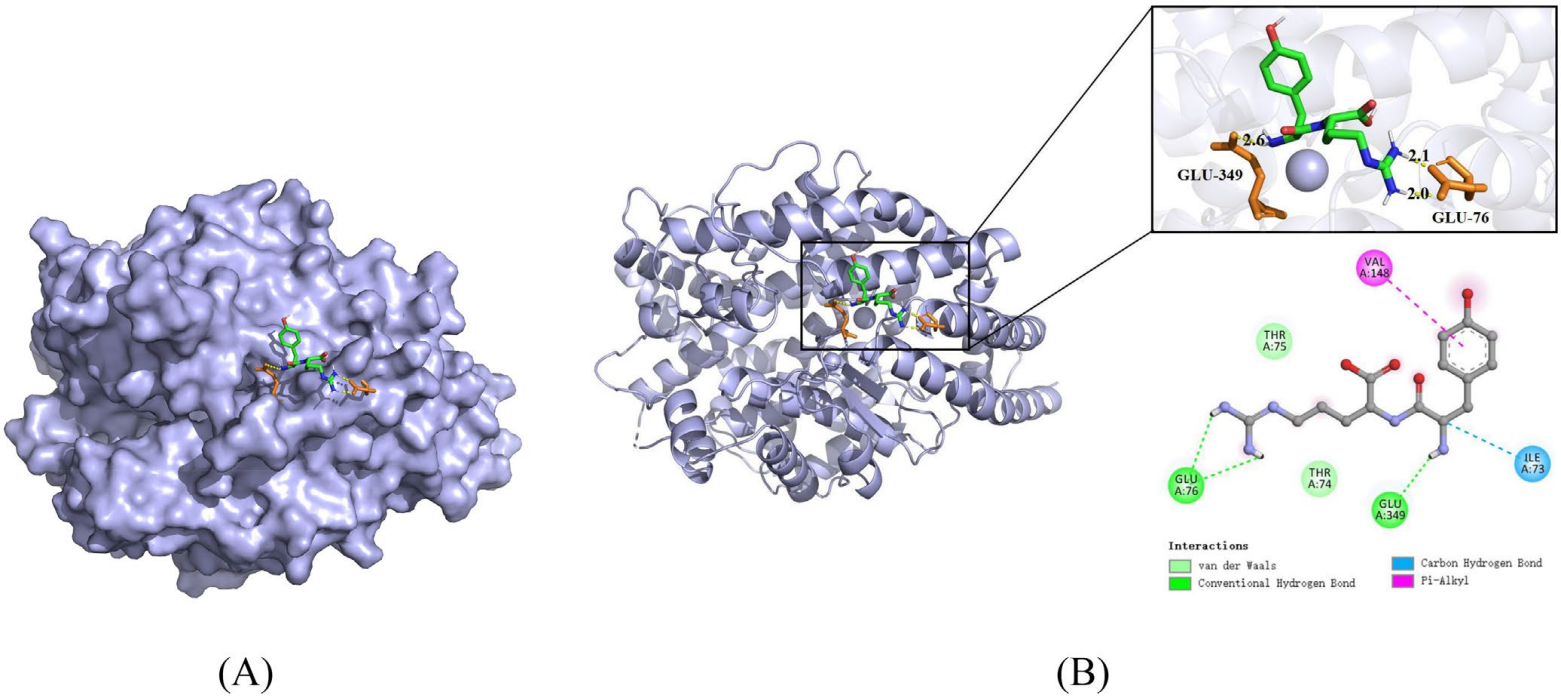
Visualization of the docking results between Tyr‐Arg and the ACE molecule. (A) Three‐dimensional binding conformation of Tyr‐Arg with ACE. (B) Three‐dimensional and two‐dimensional interaction binding patterns between Tyr‐Arg and ACE amino acid residues.

ACE contains a substrate‐binding pocket composed of residues S1 (Ala354, Glu384, Tyr523), S2 (Gln281, His353, Lys511, His513, Tyr520), and S1′ (Glu162) that are critical for substrate recognition and catalytic function (Chai et al. 2022; Chen et al. 2022; Wu et al. 2016). Molecular docking analysis unveiled that the optimal binding site for Tyr‐Arg was not located within the core catalytic site of ACE (S1, S2, S1′ pockets) but rather in a region adjacent to the active site (Figure 9A). The calculated binding free energy of −7.73 kcal/mol indicates a high binding affinity between Tyr‐Arg and ACE, which is in excellent agreement with the subsequent molecular mechanics/generalized Born surface area (MM/GBSA) result (−7.88 kcal/mol), suggesting significant inhibitory potential despite its non‐classical binding mode.

Tyr‐Arg primarily forms a stable binding complex with ACE through various noncovalent interactions, encompassing hydrogen bonds, π‐alkyl interactions, and van der Waals forces. Among these, hydrogen bonds play a pivotal role in stabilizing this interaction. Specifically, Tyr‐Arg forms two hydrogen bonds with the Glu76 residue of ACE and one hydrogen bond with the Glu349 residue, thereby contributing to binding stabilization. Furthermore, Tyr‐Arg engages in a π‐alkyl interaction with Val148 and a carbon‐hydrogen bond with Ile73. Of note, residues such as Thr74 and Thr75 interact with Tyr‐Arg via van der Waals forces, with the specific interaction patterns illustrated in Figure 9B. These initial interactions identified by docking provide the structural foundation for the complex's stability, allowing it to maintain a resilient binding conformation even during the large‐scale structural expansion observed in the 100 ns MD simulation.

The collective effect of these noncovalent interactions is synergistic, thereby facilitating the stable binding of Tyr‐Arg to ACE. The location of the Tyr‐Arg binding site outside the core catalytic site of ACE implies that Tyr‐Arg may function as a non‐competitive or allosteric inhibitor. Binding of Tyr‐Arg to the non‐catalytic region of the enzyme was found to trigger a significant domain opening movement during MD simulations. This induced conformational change is characterized by increased structural expansion (as shown by *Rg* and SASA analysis), which subsequently influences substrate binding or product release. This, in turn, results in the inhibition of enzyme activity. This mode of action is consistent with the mechanisms of action reported for certain non‐competitive or allosteric ACE inhibitors (Gao et al. 2022; Nys et al. 2016).

### 3.10 Stability Analysis of the Tyr‐Arg/ACE Complex

**FIGURE 10:**
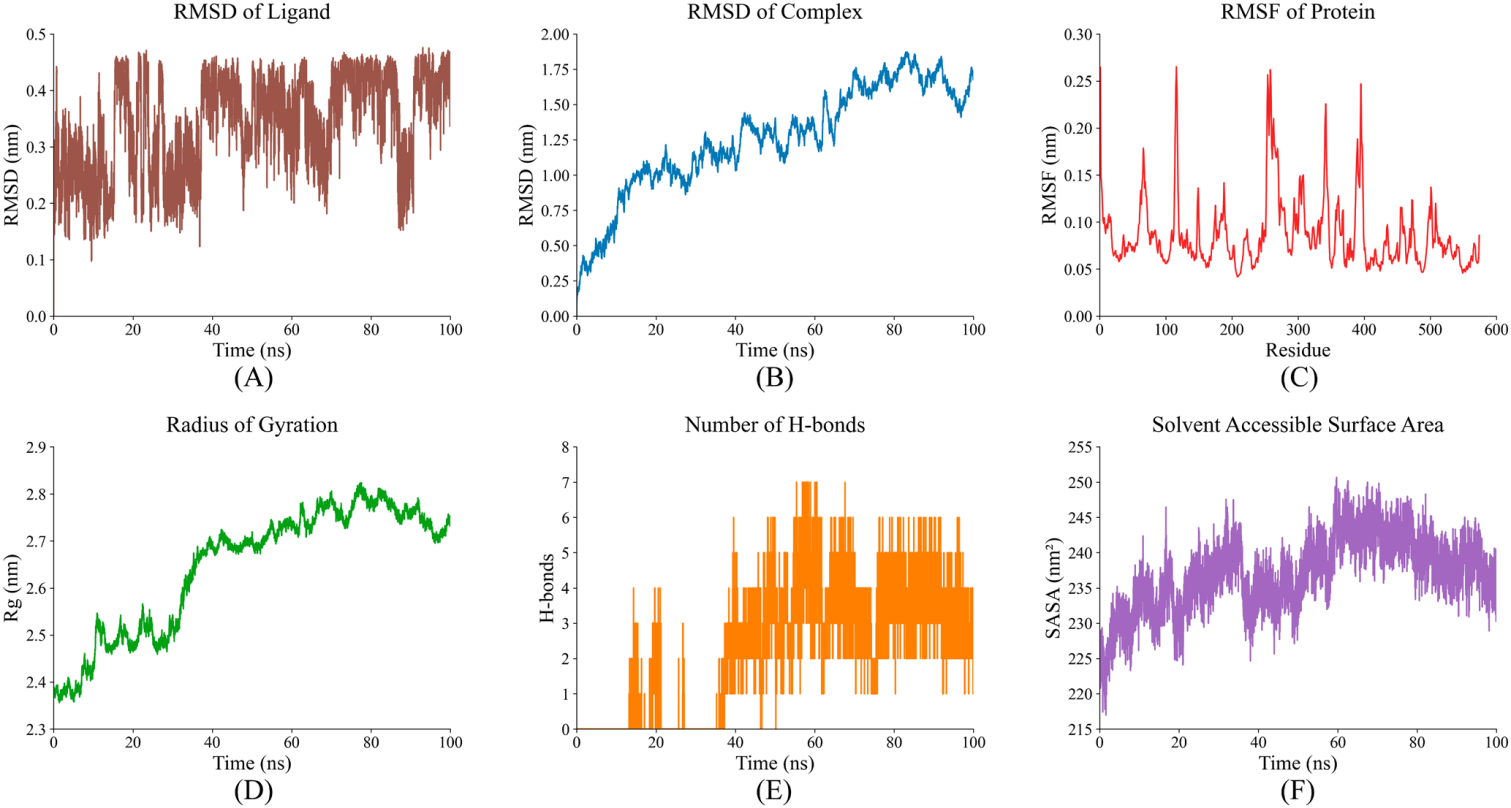
Structural stability and dynamic characteristics of the Tyr‐Arg/ACE complex during MD simulations. (A) RMSD of the Tyr‐Arg ligand. (B) RMSD of the complex. (C) RMSF of ACE protein residues. (D) R_g_ of the complex. (E) Number of intermolecular hydrogen bonds. (F) SASA of the complex.

As illustrated in Figure 10A, the ligand itself exhibited structural flexibility before stabilizing within the binding pocket. As shown in Figure 10B, RMSD analysis indicated that the Tyr‐Arg/ACE complex underwent a profound conformational adjustment phase during the initial 80 ns. The backbone RMSD of the complex reached a relative plateau of 1.5–1.8 nm after 80 ns, representing a drastic departure from the initial docking pose due to a large‐scale domain opening movement. Furthermore, as illustrated in Figure 10C, RMSF values for the protein residues were predominantly below 0.15 nm, reflecting that the protein maintained a relatively rigid core structure despite the large‐scale inter‐domain movement (Kar et al. 2013). Pronounced fluctuations (RMSF reaching 0.20–0.27 nm) were observed at residues ~120, 250–270, 350, and 400, which correspond to the protein's intrinsic flexible loops, while the binding site residues maintained a relatively stable conformation throughout the simulation.

Concomitantly, the *Rg* profile displayed an upward trend during the first 80 ns (Figure 10D), indicating that the complex underwent significant structural expansion and domain opening from the initial docking configuration before attaining a reorganized binding equilibrium. During this process, Tyr‐Arg formed 0 to 7 intermolecular hydrogen bonds with ACE, with an average of 3 to 5 during the equilibrated phase after 80 ns (Figure 10E). These persistent hydrogen‐bonding interactions provide critical energetic support for the binding affinity within the expanded state. This structural reorganization was further supported by the SASA values (Figure 10F), which reached a peak near 250 *nm* ^*2*^ between 60 and 80 ns before slightly receding and equilibrating between 235 and 245 *nm* ^*2*^ in the final 20 ns of the simulation. Collectively, these results demonstrate that the complex attained a new stable equilibrium state after extensive structural adaptation.

**FIGURE 11:**
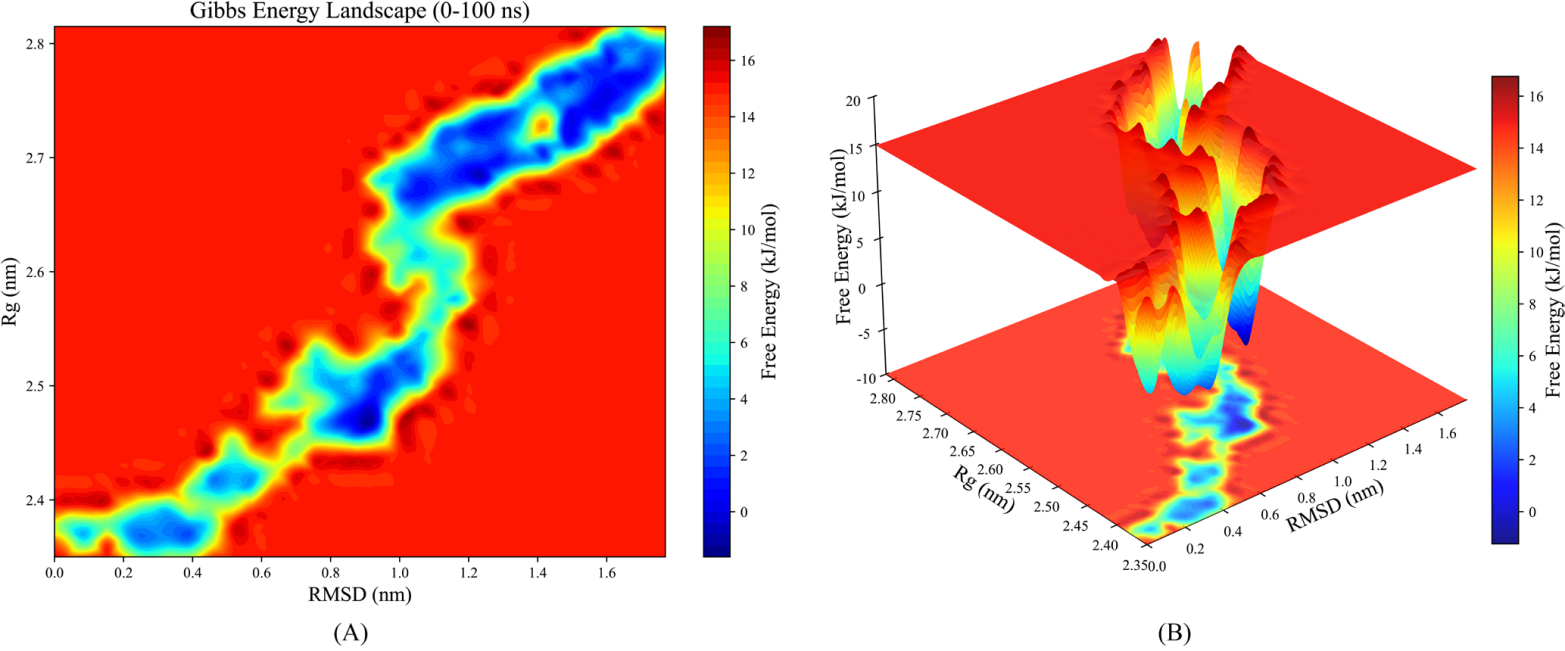
Gibbs free energy landscape (FEL) of the Tyr‐Arg/ACE complex. (A) 2D contour map and (B) 3D surface plot of the FEL projected onto RMSD and *Rg*. The color bar represents the relative free energy in kJ/mol.

As demonstrated in Figure 11A,B, the two‐dimensional (2D) free energy surface, characterized by RMSD and radius of gyration (*Rg*), along with the three‐dimensional (3D) Gibbs FEL, elucidates a step‐wise and sequential conformational transition pathway for the Tyr‐Arg/ACE complex. The complex traverses from its compact initial state through several distinct metastable intermediates (basins), ultimately settling into the global free energy minimum characterized by an expanded and open conformation at high RMSD and *Rg* values. This multi‐basin, extended pathway‐like distribution underscores that the complex undergoes substantial conformational expansion and reorganization to attain its global thermodynamic minimum, moving far from the initial docking geometry to find a more favorable energy well corresponding to the reorganized state (Okazaki et al. 2006; Shoemaker et al. 2000).

The MM/GBSA results (Table 3) indicate that the Tyr‐Arg/ACE complex exhibits a total binding free energy (Δ*G*_total_) of −7.88 kcal/mol. The exceptionally large SD (28.32 kcal/mol) is primarily attributed to the inclusion of the entire 100 ns trajectory in the sampling, thereby capturing the drastic conformational fluctuations during the initial 80 ns transition phase and the sensitive balance between large opposing magnitudes of Δ*E*_ele_ and Δ*G*_GB_ (Alanzi et al. 2025; Zhang et al. 2017). Among the individual energy components, the van der Waals interactions (Δ*E*_vdW_ = −3.59 ± 0.52 kcal/mol) and the nonpolar solvation energy (Δ*G*_surf_ = −1.01 ± 0.22 kcal/mol) exhibited minimal fluctuations. In contrast, the electrostatic energy (Δ*E*_ele_ = −168.63 ± 17.80 kcal/mol) and polar solvation energy (Δ*G*_GB_ = 165.35 ± 22.02 kcal/mol) showed significantly larger variances. This phenomenon is a characteristic manifestation of the strong coupling between polar interactions and the evolving solvent environment as the protein expands, where large opposing values largely cancel each other out (Genheden and Ryde 2011; Kollman et al. 2000). Despite these fluctuations, the average negative Δ*G*_total_ indicates a thermodynamically favorable binding process once the system reaches the equilibrium state after 80 ns.

**TABLE 3:** Binding free energy components (kcal/mol) of the Tyr‐Arg/ACE complex calculated via MM/GBSA.

Energy component	Average	SD
Δ*E*_vdW_	−3.59	0.52
Δ*E*_ele_	−168.63	17.80
Δ*G*_GB_	165.35	22.02
Δ*G*_surf_	−1.01	0.22
Δ*G*_total_	−7.88	28.32

*Note:* Δ*E*_vdW_, van der Waals energy; Δ*E*_ele_, electrostatic energy; Δ*G*_GB_, polar solvation energy; Δ*G*_surf_, nonpolar solvation energy; Δ*G*_total_, total binding free energy.

These thermodynamic results are consistent with the structural metrics, including RMSD, *Rg*, and SASA. Collectively, these findings demonstrate that although the Tyr‐Arg/ACE complex required an extended equilibration period and underwent major structural opening, it ultimately achieved a stable binding state in its reorganized form during the final 20 ns of the 100 ns simulation. The equilibrated thermodynamic state and robust binding mode suggest that although the initial docking pose necessitated drastic refinement through MD simulations, the complex ultimately converged to a stable equilibrium after 80 ns. These results underscore the critical role of large‐scale dynamic relaxation in accurately characterizing the Tyr‐Arg/ACE interaction.

## 4 Conclusion

The present study employed ultrafiltration, gel chromatography, and RP‐HPLC to successfully isolate and purify a small‐molecule peptide from Nanyang red millet Huangjiu. The peptide exhibited moderate ACE inhibitory activity and was identified as Tyr‐Arg.

Molecular docking analysis indicates that Tyr‐Arg forms a stable complex with ACE, with its binding predominantly reliant on various noncovalent interactions, such as hydrogen bonds, π‐alkyl interactions, and van der Waals forces. The synergistic effects of these interactions enhance the stability of the Tyr‐Arg complex. Notably, the binding site of Tyr‐Arg is situated outside the core catalytic site of ACE, supporting the hypothesis that Tyr‐Arg may function as a non‐competitive or allosteric inhibitor. The binding of Tyr‐Arg to the non‐catalytic region of ACE induces conformational changes in the enzyme—a phenomenon further corroborated by MD simulations, which reveal significant structural expansion—thereby impeding substrate entry into the active site or the release of products. Consequently, these alterations suppress the catalytic activity of ACE.

MD simulations, integrated with MM/GBSA calculations, were employed to evaluate the dynamic stability and energetic basis of the Tyr‐Arg/ACE complex. Trajectory analysis reveals that the complex experienced substantial conformational reorganization and structural expansion prior to attaining a quasi‐stable state after 80 ns, as demonstrated by the large changes in RMSD and *Rg*. Despite the pronounced structural fluctuations in the early and middle stages, the complex underwent notable conformational adjustments to achieve a more expanded yet energetically favorable configuration in the final 20 ns. Thermodynamic evaluation reveals that the binding affinity is collectively sustained by non‐polar interactions (van der Waals and non‐polar solvation) and a net favorable electrostatic contribution, which together yield a total binding free energy of −7.88 kcal/mol when averaged over the entire 100 ns dynamic trajectory.

The IC_50_ value of Tyr‐Arg for ACE inhibition was determined to be 158.93 ± 0.39 μM. Although this inhibitory efficacy is lower than literature‐reported standards such as Tyr‐Trp (IC_50_ = 10.50 μM) and Arg‐Phe (IC_50_ = 93.00 μM) derived from sake lees (Saito et al. 1994), Tyr‐Arg offers unique application benefits as a naturally occurring endogenous component of Nanyang red millet Huangjiu. The bioavailability of Tyr‐Arg is a critical determinant of its in vivo efficacy. Dipeptides are primarily absorbed in their intact form via the Peptide Transporter 1 (PepT1), a high‐capacity, low‐affinity transporter located on the brush border membrane of enterocytes (Smith et al. 2013). This specialized transport mechanism allows dipeptides to achieve greater bioavailability than their constituent free amino acids. For instance, dipeptides such as Gly‐Gly and Gly‐Leu are absorbed at significantly higher rates than their corresponding free amino acids in human intestinal perfusion models, suggesting that the dipeptide structure utilizes a more efficient kinetic pathway and partially bypasses the competition for amino acid transporters (Adibi 1971). Furthermore, the detection of endogenous dipeptides like Tyr‐Pro and Leu‐Lys in plasma during studies of bioactive peptides provides direct evidence of their resistance to complete hydrolysis and their successful entry into systemic circulation (Cheng et al. 2023). In summary, due to its PepT1‐mediated transport and structural stability, Tyr‐Arg demonstrates significant potential for high bioavailability, warranting further research into its physiological efficacy in vivo.

Despite the promising theoretical bioavailability discussed above, several limitations must be addressed to fully elucidate its clinical potential. Firstly, the specific transmembrane transport mechanisms of Tyr‐Arg, particularly the roles of Peptide Transporter 1 (PepT1)‐mediated transport and paracellular pathways, require detailed investigation using Caco‐2 cell models. Secondly, the lack of validation in animal models, such as spontaneously hypertensive rats (SHR), constrains the current understanding of its systemic antihypertensive effects. Accordingly, subsequent research should prioritize integrating simulated gastrointestinal digestion with in vivo pharmacokinetic and pharmacodynamic assessments to comprehensively validate the biological functions of Tyr‐Arg.

Tyr‐Arg, a bioactive dipeptide derived from natural fermentation, demonstrates exceptional biosafety and processing stability, laying a robust foundation for its application in the food industry. By isolating and purifying Tyr‐Arg from aged raw Huangjiu and subsequently reintegrating it into the wine matrix for standardized fortification, a “functional Huangjiu” with antihypertensive properties can be developed. This product development model, centered on natural bioactive peptides, provides a high‐compliance dietary intervention for the management of chronic hypertension, facilitating the transformation of traditional Huangjiu from a “sensory‐nutritional beverage” to a “precision functional food.”

This study establishes a robust technical methodology for the isolation, purification, and identification of Tyr‐Arg from Nanyang red millet Huangjiu, while elucidating the molecular binding patterns and interaction mechanisms between Tyr‐Arg and ACE via molecular docking and molecular dynamics simulations. These findings provide novel insights into the identification of bioactive peptides in Huangjiu and offer a theoretical basis for the rational design of peptide‐based functional foods, thereby highlighting the academic significance and practical potential of this study.

## Author Contributions

Conceptualization, resources, validation, project administration, J.L.; methodology, investigation, writing‐original draft preparation, X.Z.; software, data curation, visualization, writing‐review and editing, J.Z. All authors have reviewed the manuscript's final version and consented to its publication.

## Funding

This work was supported by Major Project of Collaborative Innovation in Nanyang (No. 21XTCX12005), Science and Technology Tackling Key Problems Project of Henan Province (No. 252102111046).

## Ethics Statement

The authors have nothing to report.

## Consent

The authors have nothing to report.

## Conflicts of Interest

The authors declare no conflicts of interest.

## Data Availability

The study's original data, generated and analyzed, is included in the article. For further information, please reach out to the corresponding author.

## References

[fsn371749-bib-0001] Abraham, M. J. , T. Murtola , R. Schulz , S. Páll , and E. Lindahl . 2015. “GROMACS: High Performance Molecular Simulations Through Multi‐Level Parallelism From Laptops to Supercomputers.” SoftwareX 1‐2: 19–25. 10.1016/j.softx.2015.06.001.

[fsn371749-bib-0002] Adibi, S. A. 1971. “Intestinal Transport of Dipeptides in Man: Relative Importance of Hydrolysis and Intact Absorption.” *The* Journal of Clinical Investigation 50, no. 11: 2266–2275. 10.1172/JCI106724. 5096512 PMC292168

[fsn371749-bib-0003] Ajayeoba, T. A. , S. Dula , and O. A. Ijabadeniyi . 2019. “Properties of Poly‐γ‐Glutamic Acid Producing‐Bacillus Species Isolated From Ogi Liquor and Lemon‐Ogi Liquor.” Frontiers in Microbiology 10: 771. 10.3389/fmicb.2019.00771. 31057503 PMC6481274

[fsn371749-bib-0004] Alanzi, A. R. , M. J. Alqahtani , and J. H. Alqahtani . 2025. “Computational Identification of Potential Antifungal Targets Against Claviceps Purpurea via MD Simulation and MM/GBSA.” Scientific Reports 15, no. 1: 43848. 10.1038/s41598-025-30566-5. 41326591 PMC12705770

[fsn371749-bib-0005] Arámburo‐Gálvez, J. G. , A. A. Arvizu‐Flores , F. I. Cárdenas‐Torres , et al. 2022. “Prediction of ACE‐I Inhibitory Peptides Derived From Chickpea ( *Cicer arietinum* L.): In Silico Assessments Using Simulated Enzymatic Hydrolysis, Molecular Docking and ADMET Evaluation.” Foods 11, no. 11: 1576. 10.3390/foods11111576. 35681326 PMC9180818

[fsn371749-bib-0006] Bhuyan, B. J. , and G. Mugesh . 2011. “Effect of Peptide‐Based Captopril Analogues on Angiotensin‐Converting Enzyme Activity and Peroxynitrite‐Mediated Tyrosine Nitration.” Organic & Biomolecular Chemistry 9, no. 14: 5185–5192. 10.1039/c1ob05148b. 21629912

[fsn371749-bib-0007] Chai, T. T. , C. C. Wong , M. Z. Sabri , and F. C. Wong . 2022. “Seafood Paramyosins as Sources of Anti‐Angiotensin‐Converting‐Enzyme and Anti‐Dipeptidyl‐Peptidase Peptides After Gastrointestinal Digestion: A Cheminformatic Investigation.” Molecules (Basel, Switzerland) 27, no. 12: 3864. 10.3390/molecules27123864. 35744987 PMC9229108

[fsn371749-bib-0008] Chen, H. , Y. Chen , H. Zheng , X. Xiang , and L. Xu . 2022. “A Novel Angiotensin‐I‐Converting Enzyme Inhibitory Peptide From Oyster: Simulated Gastro‐Intestinal Digestion, Molecular Docking, Inhibition Kinetics and Antihypertensive Effects in Rats.” Frontiers in Nutrition 9: 981163. 10.3389/fnut.2022.981163. 36082025 PMC9445672

[fsn371749-bib-0009] Cheng, L. , M. Tanaka , A. Yoshino , et al. 2023. “A Memory‐Improving Dipeptide, Tyr‐Pro, Can Reach the Mouse Brain After Oral Administration.” Scientific Reports 13, no. 1: 16908. 10.1038/s41598-023-44161-z. 37805661 PMC10560274

[fsn371749-bib-0010] Chingin, K. , A. Makarov , E. Denisov , O. Rebrov , and R. A. Zubarev . 2014. “Fragmentation of Positively‐Charged Biological Ions Activated With a Beam of High‐Energy Cations.” Analytical Chemistry 86, no. 1: 372–379. 10.1021/ac403193k. 24236851

[fsn371749-bib-0011] Choe, J. , B. Park , H. J. Lee , and C. Jo . 2020. “Potential Antioxidant and Angiotensin I‐Converting Enzyme Inhibitory Activity in Crust of Dry‐Aged Beef.” Scientific Reports 10, no. 1: 7883. 10.1038/s41598-020-64861-0. 32398731 PMC7217845

[fsn371749-bib-0012] Dai, H. , M. He , G. Hu , et al. 2023. “Discovery of ACE Inhibitory Peptides Derived From Green Coffee Using In Silico and In Vitro Methods.” Foods 12, no. 18: 3480. 10.3390/foods12183480. 37761189 PMC10529643

[fsn371749-bib-0013] De Pascalis, A. , A. Tomassetti , D. Vetrano , et al. 2024. “Hypertension in Cardiovascular and Kidney Disease: Recent Trends ‐ Treating Two Diseases as One.” Cardiorenal Medicine 14, no. 1: 581–587. 10.1159/000541876. 39374593

[fsn371749-bib-0014] Duan, X. , Y. Dong , M. Zhang , Z. Li , G. Bu , and F. Chen . 2023. “Identification and Molecular Interactions of Novel ACE Inhibitory Peptides From Rapeseed Protein.” Food Chemistry 422: 136085. 10.1016/j.foodchem.2023.136085. 37141758

[fsn371749-bib-0015] Feng, L. , J. Chen , W. Yan , et al. 2022. “Preparation of Active Peptides From Camellia Vietnamensis and Their Metabolic Effects in Alcohol‐Induced Liver Injury Cells.” Molecules 27, no. 6: 1790. 10.3390/molecules27061790. 35335153 PMC8951368

[fsn371749-bib-0016] Fontana, A. , F. Knuf , R. Monasterio , and A. Schieber . 2025. “Screening of Wine Industry By‐Products as a Source of Bioactive Peptides: Fractionation, In Vitro Antihypertensive Activity and Peptidomics Analysis.” Food Chemistry 476: 143478. 10.1016/j.foodchem.2025.143478. 40023130

[fsn371749-bib-0017] Gao, X. , F. Bu , D. Yi , et al. 2022. “Molecular Docking and Antihypertensive Effects of a Novel Angiotensin‐I Converting Enzyme Inhibitory Peptide From Yak Bone.” Frontiers in Nutrition 9: 993744. 10.3389/fnut.2022.993744. 36313093 PMC9605770

[fsn371749-bib-0018] Genheden, S. , and U. Ryde . 2011. “Comparison of the Efficiency of the LIE and MM/GBSA Methods to Calculate Ligand‐Binding Energies.” Journal of Chemical Theory and Computation 7, no. 11: 3768–3778. 10.1021/ct200163c. 26598269

[fsn371749-bib-0019] Giani, J. F. , L. C. Veiras , J. Z. Y. Shen , et al. 2021. “Novel Roles of the Renal Angiotensin‐Converting Enzyme.” Molecular and Cellular Endocrinology 529: 111257. 10.1016/j.mce.2021.111257. 33781839 PMC8127398

[fsn371749-bib-0020] Goyal, N. , S. N. Hajare , and S. Gautam . 2023. “Release of an Encrypted, Highly Potent ACE‐Inhibitory Peptide by Enzymatic Hydrolysis of Moth Bean ( *Vigna aconitifolia* ) Protein.” Frontiers in Nutrition 10: 1167259. 10.3389/fnut.2023.1167259. 37360301 PMC10288869

[fsn371749-bib-0021] Gu, H. , L. Liang , Z. Zhu , and X. Mao . 2022. “Preparation and Identification of Anti‐Breast Cancer Cells Peptides Released From Yak Milk Casein.” Frontiers in Nutrition 9: 997514. 10.3389/fnut.2022.997514. 36091230 PMC9462664

[fsn371749-bib-0022] He, S. , X. Mao , P. Liu , et al. 2013. “Research Into the Functional Components and Antioxidant Activities of North China Rice Wine (Ji Mo Lao Jiu).” Food Science & Nutrition 1, no. 4: 307–314. 10.1002/fsn3.39. 24804035 PMC3951597

[fsn371749-bib-0023] He, Z. , Q. Lin , Z. Qiao , et al. 2025. “Chinese Rice Wine Lees‐Derived Peptides Alleviate Hypertension by Dual Modulation of Vascular Remodeling and Mitochondrial Metabolism: Multilevel Evidence From Endothelial Cells and Hypertensive Rats.” Journal of Agricultural and Food Chemistry 73, no. 52: 33099–33111. 10.1021/acs.jafc.5c09487. 41410016

[fsn371749-bib-0024] He, Z. , Y. Zhou , S. Li , et al. 2024. “Bioactive Peptides and Evaluation of Cardiac Cytoprotective Effects of Red Millet Yellow Wine as Functional Food.” Foods 13, no. 24: 4111. 10.3390/foods13244111. 39767056 PMC11675123

[fsn371749-bib-0025] Heo, J. H. , B. H. Eom , H. W. Ryu , et al. 2020. “Acetylcholinesterase and Butyrylcholinesterase Inhibitory Activities of Khellactone Coumarin Derivatives Isolated From Peucedanum Japonicum Thurnberg.” Scientific Reports 10, no. 1: 21695. 10.1038/s41598-020-78782-5. 33303801 PMC7730441

[fsn371749-bib-0026] Homeyer, N. , and H. Gohlke . 2012. “Free Energy Calculations by the Molecular Mechanics Poisson‐Boltzmann Surface Area Method.” Molecular Informatics 31, no. 2: 114–122. 10.1002/minf.201100135. 27476956

[fsn371749-bib-0027] Huang, J. , and A. D. MacKerell Jr. 2013. “CHARMM36 All‐Atom Additive Protein Force Field: Validation Based on Comparison to NMR Data.” Journal of Computational Chemistry 34, no. 25: 2135–2145. 10.1002/jcc.23354. 23832629 PMC3800559

[fsn371749-bib-0028] Jentsch Matias de Oliveira, J. R. , M. A. Amorim , and E. André . 2020. “The Role of TRPA1 and TRPV4 Channels in Bronchoconstriction and Plasma Extravasation in Airways of Rats Treated With Captopril.” Pulmonary Pharmacology & Therapeutics 65: 102004. 10.1016/j.pupt.2021.102004. 33610768

[fsn371749-bib-0029] Jimsheena, V. K. , and L. R. Gowda . 2009. “Colorimetric, High‐Throughput Assay for Screening Angiotensin I‐Converting Enzyme Inhibitors.” Analytical Chemistry 81, no. 22: 9388–9394. 10.1021/ac901775h. 19839596

[fsn371749-bib-0030] Jin, Z. , G. Cai , C. Wu , et al. 2021. “Profiling the Key Metabolites Produced During the Modern Brewing Process of Chinese Rice Wine.” Food Research International 139: 109955. 10.1016/j.foodres.2020.109955. 33509507

[fsn371749-bib-0031] Kar, R. K. , M. Y. Ansari , P. Suryadevara , et al. 2013. “Computational Elucidation of Structural Basis for Ligand Binding With Leishmania Donovani Adenosine Kinase.” BioMed Research International 2013: 609289. 10.1155/2013/609289. 23984386 PMC3741900

[fsn371749-bib-0032] Kim, H. J. , S. G. Kang , L. Jaiswal , et al. 2016. “Identification of Four New Angiotensin I‐Converting Enzyme Inhibitory Peptides From Fermented Anchovy Sauce.” Applied Biological Chemistry 59, no. 1: 25–31. 10.1007/s13765-015-0129-4.

[fsn371749-bib-0033] Kollman, P. A. , I. Massova , C. Reyes , et al. 2000. “Calculating Structures and Free Energies of Complex Molecules: Combining Molecular Mechanics and Continuum Models.” Accounts of Chemical Research 33, no. 12: 889–897. 10.1021/ar000033j. 11123888

[fsn371749-bib-0034] Kumari, R. , R. Kumar , and A. Lynn . 2014. “g_mmpbsa–A GROMACS Tool for High‐Throughput MM‐PBSA Calculations.” Journal of Chemical Information and Modeling 54, no. 7: 1951–1962. 10.1021/ci500020m. 24850022

[fsn371749-bib-0035] Lazaridis, T. , and M. Karplus . 1999. “Effective Energy Function for Proteins in Solution.” Proteins 35, no. 2: 133–152. 10.1002/(sici)1097-0134(19990501)35:2<133::aid-prot1>3.0.co;2-n. 10223287

[fsn371749-bib-0036] Lee, J. P. , M. G. Kang , J. Y. Lee , et al. 2019. “Potent Inhibition of Acetylcholinesterase by Sargachromanol I From Sargassum Siliquastrum and by Selected Natural Compounds.” Bioorganic Chemistry 89: 103043. 10.1016/j.bioorg.2019.103043. 31200287

[fsn371749-bib-0037] Li, J. , J. Lu , C. Asakiya , et al. 2022. “Extraction and Identification of Three New *Urechis unicinctus* Visceral Peptides and Their Antioxidant Activity.” Marine Drugs 20, no. 5: 293. 10.3390/md20050293. 35621944 PMC9145011

[fsn371749-bib-0038] Li, T. , W. Du , H. Huang , et al. 2025. “Research Progress on the Mechanism of Action of Food‐Derived ACE‐Inhibitory Peptides.” Life (Basel, Switzerland) 15, no. 8: 1219. 10.3390/life15081219. 40868867 PMC12387504

[fsn371749-bib-0039] Martin, M. , and A. Deussen . 2019. “Effects of Natural Peptides From Food Proteins on Angiotensin Converting Enzyme Activity and Hypertension.” Critical Reviews in Food Science and Nutrition 59, no. 8: 1264–1283. 10.1080/10408398.2017.1402750. 29244531

[fsn371749-bib-0040] Moayedi, A. , L. Mora , M. C. Aristoy , M. Safari , M. Hashemi , and F. Toldrá . 2018. “Peptidomic Analysis of Antioxidant and ACE‐Inhibitory Peptides Obtained From Tomato Waste Proteins Fermented Using *Bacillus subtilis* .” Food Chemistry 250: 180–187. 10.1016/j.foodchem.2018.01.033. 29412909

[fsn371749-bib-0041] Nys, M. , E. Wijckmans , A. Farinha , et al. 2016. “Allosteric Binding Site in a Cys‐Loop Receptor Ligand‐Binding Domain Unveiled in the Crystal Structure of ELIC in Complex With Chlorpromazine.” Proceedings of the National Academy of Sciences of the United States of America 113, no. 43: E6696–e6703. 10.1073/pnas.1603101113. 27791038 PMC5087063

[fsn371749-bib-0042] Okazaki, K. , N. Koga , S. Takada , J. N. Onuchic , and P. G. Wolynes . 2006. “Multiple‐Basin Energy Landscapes for Large‐Amplitude Conformational Motions of Proteins: Structure‐Based Molecular Dynamics Simulations.” Proceedings of the National Academy of Sciences of the United States of America 103, no. 32: 11844–11849. 10.1073/pnas.0604375103. 16877541 PMC1567665

[fsn371749-bib-0043] Patil, U. , M. Nikoo , B. Zhang , and S. Benjakul . 2022. “Freeze‐Dried Tuna Pepsin Powder Stabilized by Some Cryoprotectants: In Vitro Simulated Gastric Digestion Toward Different Proteins and Its Storage Stability.” Foods 11, no. 15: 2292. 10.3390/foods11152292. 35954059 PMC9368244

[fsn371749-bib-0044] Puspitojati, E. , M. N. Cahyanto , Y. Marsono , and R. Indrati . 2023. “Jack Bean ( *Canavalia ensiformis* ) Tempeh: ACE‐Inhibitory Peptide Formation During Absorption in the Small Intestine.” Food Technology and Biotechnology 61, no. 1: 64–72. 10.17113/ftb.61.01.23.7635. 37200791 PMC10187565

[fsn371749-bib-0045] Qiao, Z. , J. Wang , Z. He , et al. 2022. “A Novel Angiotensin I‐Converting Enzyme Inhibitory Peptide Derived From Goat Milk Casein Hydrolysate Modulates Angiotensin II‐Stimulated Effects on Vascular Smooth Muscle Cells.” Frontiers in Nutrition 9: 878768. 10.3389/fnut.2022.878768. 35479750 PMC9037752

[fsn371749-bib-0046] Ren, N. , W. Gong , Y. Zhao , D. G. Zhao , and Y. Xu . 2022. “Innovation in Sweet Rice Wine With High Antioxidant Activity: *Eucommia ulmoides* Leaf Sweet Rice Wine.” Frontiers in Nutrition 9: 1108843. 10.3389/fnut.2022.1108843. 36704789 PMC9871602

[fsn371749-bib-0047] Saito, Y. , K. Wanezaki , A. Kawato , and S. Imayasu . 1994. “Structure and Activity of Angiotensin I Converting Enzyme Inhibitory Peptides From Sake and Sake Lees.” Bioscience, Biotechnology, and Biochemistry 58, no. 10: 1767–1771. 10.1271/bbb.58.1767. 7765503

[fsn371749-bib-0048] Shoemaker, B. A. , J. J. Portman , and P. G. Wolynes . 2000. “Speeding Molecular Recognition by Using the Folding Funnel: The Fly‐Casting Mechanism.” Proceedings of the National Academy of Sciences of the United States of America 97, no. 16: 8868–8873. 10.1073/pnas.160259697. 10908673 PMC16787

[fsn371749-bib-0049] Smith, D. E. , B. Clémençon , and M. A. Hediger . 2013. “Proton‐Coupled Oligopeptide Transporter Family SLC15: Physiological, Pharmacological and Pathological Implications.” Molecular Aspects of Medicine 34, no. 2–3: 323–336. 10.1016/j.mam.2012.11.003. 23506874 PMC3602806

[fsn371749-bib-0050] Su, Y. , S. Chen , S. Cai , et al. 2021. “A Novel Angiotensin‐I‐Converting Enzyme (ACE) Inhibitory Peptide From *Takifugu flavidus* .” Marine Drugs 19, no. 12: 651. 10.3390/md19120651. 34940650 PMC8705986

[fsn371749-bib-0051] Tang, S. , D. Chen , H. Shen , et al. 2025. “Discovery of Two Novel ACE Inhibitory Peptides From Soybeans: Stability, Molecular Interactions, and In Vivo Antihypertensive Effects.” International Journal of Biological Macromolecules 308, no. Pt 2: 142247. 10.1016/j.ijbiomac.2025.142247. 40112975

[fsn371749-bib-0052] Tran, S. , S. Kuruppu , and N. W. Rajapakse . 2022. “Chronic Renin‐Angiotensin System Activation Induced Neuroinflammation: Common Mechanisms Underlying Hypertension and Dementia?” Journal of Alzheimer's Disease: JAD 85, no. 3: 943–955. 10.3233/jad-215231. 34897090

[fsn371749-bib-0053] Wang, J. , G. Wang , Y. Zhang , R. Zhang , and Y. Zhang . 2021. “Novel Angiotensin‐Converting Enzyme Inhibitory Peptides Identified From Walnut Glutelin‐1 Hydrolysates: Molecular Interaction, Stability, and Antihypertensive Effects.” Nutrients 14, no. 1: 151. 10.3390/nu14010151. 35011025 PMC8747639

[fsn371749-bib-0054] Wang, L. , K. Qiao , Y. Huang , Y. Zhang , J. Xiao , and W. Duan . 2020. “Optimization of Beef Broth Processing Technology and Isolation and Identification of Flavor Peptides by Consecutive Chromatography and LC‐QTOF‐MS/MS.” Food Science & Nutrition 8, no. 8: 4463–4471. 10.1002/fsn3.1746. 32884726 PMC7455977

[fsn371749-bib-0055] Wei, D. , W. Fan , and Y. Xu . 2019. “In Vitro Production and Identification of Angiotensin Converting Enzyme (ACE) Inhibitory Peptides Derived From Distilled Spent Grain Prolamin Isolate.” Foods 8, no. 9: 390. 10.3390/foods8090390. 31487872 PMC6770510

[fsn371749-bib-0056] Wei, G. , F. Zhao , Z. Zhang , J. M. Regenstein , Y. Sang , and P. Zhou . 2025. “Identification and Characterization of Umami‐ACE Inhibitory Peptides From Traditional Fermented Soybean Curds.” Food Chemistry 465, no. Pt 2: 142160. 10.1016/j.foodchem.2024.142160. 39579405

[fsn371749-bib-0057] Wei, Y. , Y. Liu , Y. Li , et al. 2022. “A Novel Antihypertensive Pentapeptide Identified in Quinoa Bran Globulin Hydrolysates: Purification, In Silico Characterization, Molecular Docking With ACE and Stability Against Different Food‐Processing Conditions.” Nutrients 14, no. 12: 2420. 10.3390/nu14122420. 35745149 PMC9227351

[fsn371749-bib-0058] Wu, F. , X. Luo , Y. Zhang , et al. 2023. “Purification, Identification, and Inhibitory Mechanisms of a Novel ACE Inhibitory Peptide From Torreya Grandis.” Nutrients 15, no. 10: 2374. 10.3390/nu15102374. 37242257 PMC10224335

[fsn371749-bib-0059] Wu, Q. , J. Du , J. Jia , and C. Kuang . 2016. “Production of ACE Inhibitory Peptides From Sweet Sorghum Grain Protein Using Alcalase: Hydrolysis Kinetic, Purification and Molecular Docking Study.” Food Chemistry 199: 140–149. 10.1016/j.foodchem.2015.12.012. 26775955

[fsn371749-bib-0060] Zeng, W. , X. Yu , M. Chen , et al. 2025. “ACE‐Inhibitory Peptides From Morchella Esculenta: Screening, Kinetics, and Molecular Dynamics Simulation.” Food Chemistry 490: 145011. 10.1016/j.foodchem.2025.145011. 40499431

[fsn371749-bib-0061] Zhang, X. , H. Perez‐Sanchez , and F. C. Lightstone . 2017. “A Comprehensive Docking and MM/GBSA Rescoring Study of Ligand Recognition Upon Binding Antithrombin.” Current Topics in Medicinal Chemistry 17, no. 14: 1631–1639. 10.2174/1568026616666161117112604. 27852201 PMC5403970

[fsn371749-bib-0062] Zheng, Y. , Y. Zhang , and S. San . 2020. “Efficacy of a Novel ACE‐Inhibitory Peptide From Sargassum Maclurei in Hypertension and Reduction of Intracellular Endothelin‐1.” Nutrients 12, no. 3: 653. 10.3390/nu12030653. 32121212 PMC7146574

[fsn371749-bib-0063] Zhou, J. , Q. Han , T. Koyama , and S. Ishizaki . 2023. “Preparation, Purification and Characterization of Antibacterial and ACE Inhibitory Peptides From Head Protein Hydrolysate of Kuruma Shrimp, *Marsupenaeus japonicus* .” Molecules 28, no. 2: 894. 10.3390/molecules28020894. 36677951 PMC9861681

[fsn371749-bib-0064] Zhuang, M. , L. Lin , M. Zhao , et al. 2016. “Sequence, Taste and Umami‐Enhancing Effect of the Peptides Separated From Soy Sauce.” Food Chemistry 206: 174–181. 10.1016/j.foodchem.2016.03.058. 27041313

